# Sociodemographic disparities and reasons for delayed healthcare among U.S. cancer survivors: an All of Us study

**DOI:** 10.1007/s00520-026-10880-y

**Published:** 2026-06-16

**Authors:** Ding Quan Ng, Zhiyuan Zheng, Ahmedin Jemal, Alexandre Chan, Farhad Islami

**Affiliations:** 1 Department of Clinical Pharmacy Practice, School of Pharmacy & Pharmaceutical Sciences, University of California Irvine, 802 W Peltason Dr, Irvine, CA 92697 USA; 2 Surveillance, Prevention, and Health Services Research, American Cancer Society, Atlanta, GA USA; 3 Section of Medical Oncology & Hematology, Department of Internal Medicine, Yale School of Medicine & Yale Cancer Center, New Haven, CT USA

**Keywords:** Cancer survivors, Delayed healthcare, Healthcare access, Survivorship care, Health disparity

## Abstract

**Objective:**

To examine how sociodemographic factors influence healthcare access among cancer survivors.

**Methods:**

From the National Institutes of Health’s All of Us dataset (2018–2022, *n* = 27,589), we analyzed the relationship between characteristics like age, income, race/ethnicity, and insurance, and reasons for delayed healthcare, including affordability, transportation, and nervousness.

**Results:**

Young adult cancer survivors (ages 18–39), those on Medicaid, and individuals earning less than $25,000 annually consistently experienced higher rates of delayed healthcare. The top reasons for delayed healthcare were affordability issues (12%), nervousness (8%), and transportation barriers (6%). Female survivors were more likely to delay care for all reasons except transportation. Work and caregiving-related delays were more common among minoritized racial/ethnic groups, while non-Hispanic White survivors more often delayed healthcare due to nervousness and socioeconomic factors.

**Conclusions:**

Considerable differences in delayed healthcare were observed among cancer survivors by sociodemographic characteristics. Findings highlight the need for tailored interventions to effectively address the unique social needs of each cancer survivor, ultimately improving healthcare access for all.

**Supplementary Information:**

The online version contains supplementary material available at 10.1007/s00520-026-10880-y.

## Introduction

Across the survivorship care continuum, cancer patients require consistent and timely healthcare engagement to navigate complex treatment plans, address cancer-specific needs such as treatment toxicities and oncofertility, and adhere to rigorous surveillance protocols necessary to monitor for disease recurrence [1]. Delayed and inadequate survivorship follow-up care can lead to poorer survival, wellness, and quality of life, adoption of adverse lifestyle behaviors, late detection of recurrences, and suboptimal control of complications, such as cardiomyopathy, fatigue, pain, neuropathy, psychological distress, and neurocognitive impairments [2–6]. In a survey conducted by the Multinational Association of Supportive Care in Cancer, timely supportive care is identified by cancer practitioners as the top practice-related disparity that is experienced by cancer patients in the United States (U.S.) [7].

There are many reasons why cancer survivors would forgo or delay medical care. High out-of-pocket costs and medical financial hardships are most frequently investigated as they directly affect patient’s ability to pay for healthcare services, especially for individuals with lower incomes and without health insurance [8–11]. Other barriers include lack of sick leave from workplace, family responsibilities, lack of transportation, and fear of stigma [12–15]. The effects of each barrier to healthcare are multidimensional, as each may interplay with other demographic, social, economic, and cultural factors. As such, tailoring patient navigation strategies to an individual’s social needs is likely to be more effective in addressing healthcare access barriers [16].

Previous studies have examined disparities in health-related social needs, medical financial hardship, and health outcomes among cancer survivors [17–19]. Few studies, however, have evaluated disparities across a variety of potential reasons for delayed healthcare, as a function of patient-level characteristics and their interactions, with adequate statistical power. Understanding these aspects of access-to-care barriers can help provide tailored care to cancer survivors.

**Fig. 1:**
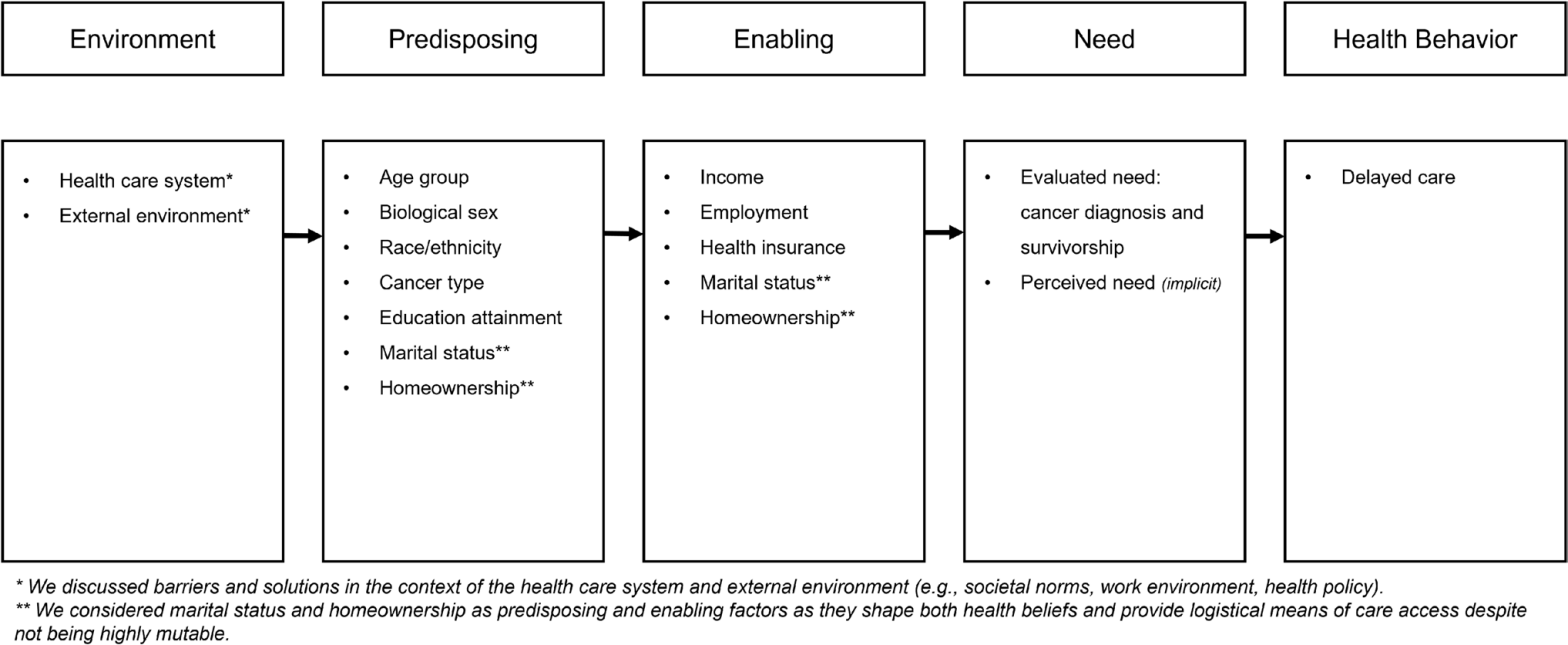
The adapted behavioral model for health services utilization adapted from the Andersen’s model

To address this knowledge gap, we aimed to comprehensively evaluate the association of sociodemographic characteristics with major reasons for delayed healthcare among cancer survivors. We adapted the Andersen’s Behavioral Model of Health Services Use [20], which organizes determinants of healthcare utilization into predisposing characteristics, enabling resources, and perceived need (Fig. 1), to discuss our findings. Situating our variables within this framework provides a conceptual basis for interpreting disparities and identifying intervention targets across multiple domains of delayed healthcare. Furthermore, we performed stratified analyses to provide a preliminary investigation of intersectionality within our findings.

## Methods

### Dataset

The All of Us Research Program has been administered by the National Institutes of Health (NIH) since May 2018 to facilitate biomedical research in accordance with the Precision Medicine Initiative launched in 2015 [21, 22]. The study was conducted cross all U.S. states, although enrollment density varied by region [23]. The surveys were developed through an iterative process that involved prioritization of scientific domains, survey development with available validated instruments, survey refinement with cognitive interviews and online testing, and survey review and approval by key stakeholders [24]. For this study, we used data from the All of Us Controlled Tier Dataset version 7 (C2022Q4R9).

### Participants

Eligible participants for the current study included those who completed the “Health Care Access & Utilization” survey and were diagnosed with cancer, either self-reported (“Overall Health” survey) or with ≥ 2 EHR diagnosis records of cancer to reduce likelihood of false-positive cancer diagnoses (ICD-9-CM: 140 to 209, ICD-10-CM: C00 to C96) prior to survey completion. Participants who were diagnosed with only skin cancer were excluded as the majority were likely non-melanoma cancers with relatively minor survivorship care needs [25, 26].

### Covariates

Data on self-reported biological sex, race/ethnicity, educational attainment, annual household income, employment, health insurance, marital status, and homeownership status were obtained from the “Baseline” survey [27]. Based on the date of completion of the outcome questionnaire in that survey and self-reported date of birth, participants’ age at the time of survey was calculated. All research data are organized in the Observational Health and Medicines Outcomes Partnership (OMOP) common data model v5.2 [28].

### Outcomes

Within the “Health Care Access & Utilization” survey, participants were also asked about delayed healthcare: “*There are many reasons people delay getting medical care. Have you delayed getting care for any of the following reasons in the past 12 months*?” These reasons included “*could not afford co-pay, could not afford deductible, need of out-of-pocket payment, could not get time off work, provided care to an adult, could not get childcare, did not have transportation, and were nervous about seeing a provider*”. For each listed reason for delayed healthcare, the following responses were made available: “Yes”, “No” or “Don’t know”. We combined reasons related to out-of-pocket payment, co-pay, and deductible expenditure into one category as “affordability”.

### Statistical analysis

The distribution of sociodemographic characteristics was presented with overall counts and percentages, stratified by reasons for delayed healthcare. Using logistic regression, associations between evaluated sociodemographic characteristics and reasons for delayed healthcare were examined, overall and after stratification by age group (18–39, 40–64, 65 + years), biological sex (female, male), race/ethnicity (non-Hispanic White [NHW], non-Hispanic Black [NHB], Hispanic/Latinx), and cancer type (four leading causes of cancer deaths [29]: breast [female only], prostate, colorectal, lung). Group differences between strata were evaluated with Z-test for comparing two independent regression coefficients $\left(Z=\frac{{{b}_{1}-b}_{2}}{\sqrt{{{SEb}_{1}^{2}-SEb}_{2}^{2}}}\right)$ [30, 31]. In this formula, b_1_ and b_2_ represent the regression coefficients from two independent strata, and SEb_1_ and SEb_2_ are their respective standard errors. Effect sizes were presented as odds ratios (OR) and 95% confidence intervals (CI) with the largest category serving as the reference group for each categorical characteristic. Statistical significance in the adjusted models with all cancer survivors were determined by a Bonferroni-corrected significance level of 0.000397 *[0.05/(21 covariates* × *6 reasons for delayed healthcare)]*. All other analyses were similarly two-tailed but tested at 5% significance level. We accessed the data and performed all analyses using R (version 4.1.0) in a cloud-based platform operated on Jupyter Notebook.

## Results

Of 413,360 participants in the All of Us Controlled Tier Dataset version 7 through July 1 st, 2022, a total of 27,589 cancer survivors were included in this analysis (eFigure S1), of which 6,624 (24%) reported at least one reason for delayed healthcare (Table 1). The majority of cancer survivors were 65 years or older (55.7%), female (61.9%), identified as NHW (81.9%), attained above high school education (88.4%), had private health insurance (57.4%), and were married (59.9%) and homeowners (74.7%) (Table 1).

**Table 1:** Sociodemographic characteristics among cancer survivors in the All of Us Research Program, stratified by reasons for delayed healthcare

Variable	*Overall (N* = *27,589)*	Reasons for delayed healthcare in the past 12 months						
		≥ 1 reason(s) for delayed healthcare (*N* = 6624)	Affordability^1^ (*N* = 3500)	Work (*N* = 1271)	Elderly care (*N* = 433)	Childcare (*N* = 324)	Transportation (*N* = 1540)	Nervous (*N* = 2282)
Prevalence, %	***100%***	**24%**	**12.7%**	**4.6%**	**1.6%**	**1.2%**	**5.6%**	**8.3%**
Age at survey, *n* (%^3^)								
18–39	*1541 (5.6%)*	880 (57.1%)	439 (28.5%)	316 (20.5%)	32 (2.1%)	165 (10.7%)	227 (14.7%)	396 (25.7%)
40–64	*10,686 (38.7%)*	3702 (34.6%)	2025 (19.0%)	835 (7.8%)	254 (2.4%)	NR^4^	872 (8.2%)	1246 (11.7%)
65 +	*15,362 (55.7%)*	2042 (13.3%)	1036 (6.7%)	120 (0.8%)	147 (1.0%)	≤ 20	441 (2.9%)	640 (4.2%)
Biological sex, *n* (%^3^)								
Male	*10,196 (37.9%)*	1677 (16.5%)	905 (8.9%)	231 (2.3%)	86 (0.8%)	36 (0.4%)	415 (4.1%)	504 (4.9%)
Female	*16,651 (61.9%)*	4734 (28.4%)	2468 (14.8%)	993 (6.0%)	333 (2.0%)	274 (1.6%)	1065 (6.4%)	1706 (10.2%)
Race/ethnicity, *n* (%^3^)								
NH White	*21,765 (81.9%)*	4780 (22%)	2547 (11.7%)	878 (4.0%)	283 (1.3%)	191 (0.9%)	970 (4.5%)	1729 (7.9%)
NH Black	*1851 (7.0%)*	610 (33%)	311 (16.8%)	100 (5.4%)	46 (2.5%)	31 (1.7%)	235 (12.7%)	169 (9.1%)
Hispanic/Latinx	*1475 (5.6%)*	488 (33.1%)	253 (17.2%)	121 (8.2%)	43 (2.9%)	41 (2.8%)	137 (9.3%)	145 (9.8%)
NH Asian/NHPI	*461 (1.7%)*	119 (25.8%)	54 (11.7%)	33 (7.2%)	≤ 20	≤ 20	23 (5.0%)	30 (6.5%)
Educational attainment, *n* (%^3^)								
High school diploma or less	*3125 (11.6%)*	991 (31.7%)	471 (15.1%)	143 (4.6%)	79 (2.5%)	55 (1.8%)	386 (12.4%)	343 (11.0%)
Some college	*6624 (24.7%)*	1923 (29%)	1082 (16.3%)	362 (5.5%)	136 (2.1%)	94 (1.4%)	527 (8.0%)	652 (9.8%)
Bachelor	*7552 (28.1%)*	1779 (23.6%)	959 (12.7%)	378 (5.0%)	101 (1.3%)	86 (1.1%)	327 (4.3%)	614 (8.1%)
Master or more	*9558 (35.6%)*	1729 (18.1%)	874 (9.1%)	351 (3.7%)	102 (1.1%)	78 (0.8%)	241 (2.5%)	610 (6.4%)
Annual household income, *n* (%^3^)								
Less than $25,000	*3335 (12.1%)*	1415 (42.4%)	666 (20.0%)	148 (4.4%)	127 (3.8%)	75 (2.2%)	675 (20.2%)	504 (15.1%)
$25,000—$49,999	*4079 (14.8%)*	1212 (29.7%)	749 (18.4%)	240 (5.9%)	88 (2.2%)	58 (1.4%)	276 (6.8%)	358 (8.8%)
$50,000—$99,999	*7247 (26.3%)*	1571 (21.7%)	901 (12.4%)	338 (4.7%)	92 (1.3%)	75 (1.0%)	209 (2.9%)	526 (7.3%)
$100,000—$199,999	*6266 (22.7%)*	1126 (18%)	579 (9.2%)	286 (4.6%)	46 (0.7%)	50 (0.8%)	104 (1.7%)	428 (6.8%)
$200,000 and above	*2903 (10.5%)*	421 (14.5%)	167 (5.8%)	117 (4.0%)	≤ 20	27 (0.9%)	41 (1.4%)	170 (5.9%)
Missing	*3759 (13.6%)*	879 (23.4%)	438 (11.7%)	142 (3.8%)	NR^4^	39 (1.0%)	235 (6.3%)	296 (7.9%)
Employment status, *n* (%^3^)								
Employed	*10,359 (38.6%)*	3054 (29.5%)	1701 (16.4%)	1064 (10.3%)	139 (1.3%)	153 (1.5%)	368 (3.6%)	1010 (9.7%)
Not working	*16,479 (61.4%)*	3358 (20.4%)	1685 (10.2%)	174 (1.1%)	277 (1.7%)	157 (1.0%)	1109 (6.7%)	1201 (7.3%)
Health insurance, *n* (%^3^)								
Private	*15,415 (57.4%)*	3594 (23.3%)	2009 (13.0%)	958 (6.2%)	174 (1.1%)	166 (1.1%)	452 (2.9%)	1263 (8.2%)
Medicare/Dual eligibility	*8208 (30.5%)*	1503 (18.3%)	780 (9.5%)	67 (0.8%)	119 (1.4%)	26 (0.3%)	499 (6.1%)	470 (5.7%)
Medicaid	*1840 (6.9%)*	844 (45.9%)	305 (16.6%)	124 (6.7%)	90 (4.9%)	95 (5.2%)	396 (21.5%)	340 (18.5%)
Uninsured (including IHS only, single service plans)	*388 (1.4%)*	190 (49%)	140 (36.1%)	31 (8.0%)	13 (3.4%)	≤ 20	55 (14.2%)	55 (14.2%)
Marital status, *n* (%^3^)								
Married	*16,078 (59.9%)*	3150 (19.6%)	1729 (10.8%)	619 (3.8%)	187 (1.2%)	185 (1.2%)	456 (2.8%)	1101 (6.8%)
Divorced/Separated/Widowed	*6719 (25.0%)*	1840 (27.4%)	957 (14.2%)	292 (4.3%)	135 (2.0%)	60 (0.9%)	598 (8.9%)	575 (8.6%)
Non-married	*2981 (11.1%)*	1045 (35.1%)	512 (17.2%)	228 (7.6%)	73 (2.4%)	40 (1.3%)	336 (11.3%)	393 (13.2%)
Living with partner	*1062 (4.0%)*	368 (34.7%)	181 (17.0%)	91 (8.6%)	21 (2.0%)	28 (2.6%)	82 (7.7%)	138 (13.0%)
Homeownership, *n* (%^3^)								
Owner	*19,934 (74.7%)*	3810 (19.1%)	2050 (10.2%)	698 (3.5%)	237 (1.2%)	134 (0.7%)	570 (2.9%)	1314 (6.6%)
Renter	*466 (20.5%)*	2061 (37.7%)	1064 (19.5%)	430 (7.9%)	116 (2.1%)	139 (2.5%)	701 (12.8%)	691 (12.6%)
Cancer types^2^, *n* (%^3^)								
Breast (female only)	*5566 (20.2%)*	1327 (23.8%)	685 (12.3%)	253 (4.5%)	94 (1.7%)	62 (1.1%)	226 (4.1%)	468 (8.4%)
Prostate	*2822 (10.2%)*	366 (13%)	211 (7.5%)	30 (1.1%)	≤ 20	≤ 20	72 (2.6%)	102 (3.6%)
Colorectal	*943 (3.4%)*	241 (25.6%)	130 (13.8%)	38 (4.0%)	≤ 20	≤ 20	66 (7.0%)	93 (9.9%)
Lung	*535 (1.9%)*	108 (20.2%)	49 (9.2%)	≤ 20	≤ 20	≤ 20	30 (5.6%)	36 (6.7%)

*NH* non-Hispanic; *NR* not reported; *NHPI* Native Hawaiian, Pacific Islander

^1^Delayed healthcare due to affordability reasons includes: could not afford co-pay, could not afford deductible, and need of out-of-pocket payment

^2^Exclude multiple cancers

^3^Row percentages are presented for all columns other than the “Overall” column, where they present column percentages

^4^NR cells are masked even though they have > 20 counts to prevent the back-calculation of cells with ≤ 20 counts in compliance with the All of Us Data and Statistics Dissemination Policy

### Reasons for delayed healthcare

The top reasons for delayed healthcare were affordability problems with out-of-pocket healthcare expenditure (12.4%), nervousness about seeing a provider (8.3%), and lack of transportation means (5.6%) (Table 1). Cancer survivors aged 18–39 years, enrolled in Medicaid, and earning less than $25,000 a year reported most likely to report delayed healthcare (Table 1), and were consistently among the top 3 strata with the highest prevalence of delayed healthcare across the evaluated reasons (Table 2).

**Table 2:** Top three sociodemographic characteristics with highest prevalence of delayed healthcare for each reason among cancer survivors in the All of Us Research Program

Reasons for delayed healthcare in the past 12 months	Overall prevalence, %	Prevalence Rank 1	Prevalence Rank 2	Prevalence Rank 3
		Subgroup, %		
Affordability^1^	12.7%	Age: 18–39 (28.5%)	Income: < $25 k (20.0%)	Homeownership: Renter (19.5%)
Work	4.6%	Age: 18–39 (20.5%)	Employment: Employed (10.3%)	Marital status: Living with partner (8.6%)
Elderly care	1.6%	Insurance: Medicaid (4.9%)	Income: < $25 k (3.8%)	Insurance: Uninsured (3.4%)
Childcare	1.2%	Age: 18–39 (10.7%)	Insurance: Medicaid (5.2%)	Race/ethnicity: Hispanic (2.8%)
Transportation	5.6%	Insurance: Medicaid (21.5%)	Income: < $25 k (20.2%)	Age: 18–39 (14.7%)
Nervous	8.3%	Age: 18–39 (25.7%)	Insurance: Medicaid (18.5%)	Income: < $25 k (15.1%)

^1^Delayed healthcare due to affordability reasons includes: could not afford co-pay, could not afford deductible, and need of out-of-pocket payment

In Bonferroni-corrected adjusted analyses of the full cohort, delayed healthcare was associated with age at survey (due to all 6 reasons), biological sex (all reasons except transportation), health insurance (all reasons except nervousness), income (affordability, transportation, and nervousness), employment and homeownership status (affordability, work, and transportation), marital status (childcare and transportation), and race/ethnicity (nervousness). In contrast, delayed healthcare was not associated with education attainment for any reason. Table 3 details these findings, which are summarized in eTable S1.

**Table 3:** Odds ratios (95% confidence intervals) for the association between sociodemographic factors and delayed healthcare among cancer survivors in the All of Us Research Program

	Affordability^1^		Work		Elderly care		Childcare		Transportation		Nervous	
	Unadj	Adj	Unadj	Adj	Unadj	Adj	Unadj	Adj	Unadj	Adj	Unadj	Adj
Age at survey												
18–39	4.58 (4.03–5.21)	3.79^*^ **(3.20–4.46)**	29.78 (23.93–37.05)	8.33^*^ **(6.38–10.89)**	1.83 (1.24–2.69)	1.13 (0.70–1.83)	90.94 (55.02–150.30)	70.89^*^ **(38.43–130.78)**	5.78 (4.88–6.85)	5.19^*^ **(4.11–6.56)**	7.54 (6.56–8.67)	5.59^*^ **(4.66–6.71)**
40–64	2.95 (2.72–3.20)	2.78^*^ **(2.51–3.09)**	10.16 (8.37–12.32)	3.71^*^ **(2.96–4.65)**	2.28 (1.86–2.80)	1.86^*^ **(1.42–2.44)**	11.01 (6.65–18.22)	8.34^*^ **(4.66–14.93)**	3.00 (2.67–3.38)	2.99^*^ **(2.56–3.48)**	2.94 (2.66–3.24)	2.48^*^ **(2.18–2.81)**
65 +	1.00	1.00	1.00	1.00	1.00	1.00	1.00	1.00	1.00	1.00	1.00	1.00
Biological sex												
Female	1.00	1.00	1.00	1.00	1.00	1.00	1.00	1.00	1.00	1.00	1.00	1.00
Male	0.57 (0.53–0.62)	0.79^*^ **(0.72–0.86)**	0.37 (0.32–0.42)	0.62^*^ **(0.53–0.73)**	0.43 (0.34–0.54)	0.52^*^ **(0.40–0.67)**	0.22 (0.15–0.31)	0.31^*^ **(0.21–0.47)**	0.62 (0.55–0.69)	0.92 (0.80–1.05)	0.45 (0.41–0.50)	0.59^*^ **(0.52–0.66)**
Race/ethnicity												
NH-White	1.00	1.00	1.00	1.00	1.00	1.00	1.00	1.00	1.00	1.00	1.00	1.00
NH-Black	1.59 (1.39–1.81)	1.10 (0.95–1.28)	1.42 (1.14–1.75)	1.00 (0.78–1.29)	1.99 (1.45–2.74)	1.08 (0.75–1.55)	1.99 (1.36–2.92)	1.40 (0.87–2.23)	3.18 (2.73–3.70)	1.17 (0.97–1.41)	1.22 (1.03–1.44)	0.74 (0.61–0.89)
Hispanic	1.53 (1.33–1.77)	0.86 (0.73–1.02)	2.14 (1.75–2.60)	1.17 (0.93–1.49)	2.25 (1.62–3.12)	1.14 (0.77–1.70)	3.23 (2.29–4.55)	1.52 (1.01–2.27)	2.25 (1.86–2.71)	0.87 (0.70–1.09)	1.28 (1.07–1.53)	0.66^*^ **(0.53–0.81)**
NH-Asian/NHPI	0.98 (0.74–1.32)	0.75 (0.54–1.03)	1.81 (1.26–2.60)	1.16 (0.78–1.72)	1.50 (0.76–2.93)	1.61 (0.81–3.19)	3.00 (1.66–5.43)	1.83 (0.92–3.62)	1.13 (0.74–1.73)	0.92 (0.56–1.52)	0.80 (0.55–1.16)	0.54 (0.35–0.81)
Education attainment												
High school diploma or less	1.80 (1.59–2.03)	0.99 (0.85–1.14)	1.30 (1.06–1.58)	1.11 (0.88–1.41)	2.44 (1.81–3.28)	0.97 (0.68–1.38)	2.21 (1.56–3.13)	0.67 (0.42–1.05)	5.58 (4.72–6.59)	1.42 (1.15–1.74)	1.89 (1.64–2.17)	1.10 (0.93–1.31)
Some college	1.96 (1.78–2.16)	1.19 (1.07–1.34)	1.54 (1.33–1.79)	1.25 (1.05–1.49)	1.95 (1.50–2.52)	0.96 (0.72–1.29)	1.75 (1.30–2.37)	0.84 (0.54–1.31)	3.37 (2.88–3.93)	1.34 (1.12–1.61)	1.63 (1.45–1.83)	1.05 (0.92–1.21)
Bachelor	1.43 (1.29–1.57)	1.09 (0.98–1.22)	1.38 (1.19–1.60)	1.08 (0.92–1.28)	1.23 (0.93–1.63)	0.89 (0.67–1.20)	1.38 (1.01–1.87)	0.89 (0.64–1.25)	1.75 (1.48–2.07)	1.16 (0.96–1.40)	1.31 (1.16–1.47)	1.06 (0.92–1.20)
Master or more	1.00	1.00	1.00	1.00	1.00	1.00	1.00	1.00	1.00	1.00	1.00	1.00
Annual household income												
Less than $25,000	1.78 (1.59–1.99)	1.62^*^ **(1.39–1.88)**	0.97 (0.80–1.19)	1.25 (0.96–1.64)	3.09 (2.35–4.06)	1.78 (1.24–2.55)	2.22 (1.60–3.06)	0.84 (0.54–1.31)	8.73 (7.42–10.26)	2.78^*^ **(2.26–3.42)**	2.36 (2.08–2.69)	1.67^*^ **(1.40–1.99)**
$25,000—$49,999	1.63 (1.47–1.82)	1.62^*^ **(1.44–1.83)**	1.32 (1.12–1.57)	1.30 (1.06–1.59)	1.75 (1.30–2.35)	1.52 (1.10–2.08)	1.41 (1.00–1.99)	0.92 (0.62–1.38)	2.47 (2.05–2.97)	1.59^*^ **(1.30–1.94)**	1.26 (1.09–1.45)	1.12 (0.96–1.31)
$50,000—$99,999	1.00	1.00	1.00	1.00	1.00	1.00	1.00	1.00	1.00	1.00	1.00	1.00
$100,000—$199,999	0.69 (0.62–0.77)	0.62^*^ **(0.55–0.70)**	0.95 (0.81–1.12)	0.83 (0.70–1.00)	0.56 (0.39–0.79)	0.56 (0.38–0.81)	0.75 (0.52–1.07)	0.68 (0.46–1.01)	0.56 (0.44–0.72)	0.68 (0.53–0.87)	0.91 (0.80–1.04)	0.93 (0.80–1.07)
$200,000 and above	0.41 (0.35–0.49)	0.35^*^ **(0.29–0.43)**	0.84 (0.68–1.04)	0.70 (0.55–0.89)	0.31 (0.17–0.58)	0.32 (0.17–0.60)	0.88 (0.56–1.37)	0.96 (0.59–1.57)	0.48 (0.34–0.67)	0.66 (0.46–0.94)	0.77 (0.64–0.92)	0.79 (0.65–0.95)
Employment status												
Employed	1.00	1.00	1.00	1.00	1.00	1.00	1.00	1.00	1.00	1.00	1.00	1.00
Not working	0.62 (0.57–0.66)	0.76^*^ **(0.69–0.84)**	0.10 (0.08–0.11)	0.16^*^ **(0.13–0.20)**	1.34 (1.09–1.65)	1.15 (0.90–1.46)	0.68 (0.55–0.85)	1.50 (1.13–1.99)	1.98 (1.76–2.23)	1.86^*^ **(1.60–2.16)**	0.75 (0.69–0.82)	1.03 (0.92–1.15)
Health insurance												
Private	1.00	1.00	1.00	1.00	1.00	1.00	1.00	1.00	1.00	1.00	1.00	1.00
Medicare/Dual eligibility	0.74 (0.67–0.80)	1.00 (0.89–1.12)	0.13 (0.10–0.17)	0.53^*^ **(0.39–0.72)**	1.36 (1.08–1.72)	1.38 (1.03–1.85)	0.31 (0.20–0.47)	1.03 (0.61–1.73)	2.16 (1.89–2.46)	1.56^*^ **(1.32–1.85)**	0.70 (0.63–0.78)	0.95 (0.83–1.10)
Medicaid	1.31 (1.14–1.49)	0.54^*^ **(0.46–0.64)**	1.10 (0.91–1.34)	0.73 (0.56–0.94)	4.46 (3.44–5.79)	1.94^*^ **(1.37–2.74)**	4.98 (3.85–6.44)	2.38^*^ **(1.61–3.52)**	9.26 (8.01–10.72)	1.49^*^ **(1.23–1.81)**	2.60 (2.28–2.97)	1.10 (0.92–1.31)
Uninsured (including IHS only, single service plans)	3.99 (3.19–4.98)	2.07^*^ **(1.59–2.69)**	1.32 (0.91–1.92)	0.82 (0.52–1.28)	3.07 (1.73–5.45)	1.80 (0.96–3.37)	3.46 (1.98–6.04)	2.70 (1.40–5.21)	5.62 (4.16–7.60)	1.59 (1.12–2.26)	1.91 (1.42–2.55)	1.15 (0.82–1.61)
Marital status												
Married	1.00	1.00	1.00	1.00	1.00	1.00	1.00	1.00	1.00	1.00	1.00	1.00
Divorced/Separated/Widowed	1.42 (1.31–1.55)	0.95 (0.86–1.06)	1.17 (1.01–1.35)	1.12 (0.94–1.33)	1.79 (1.43–2.23)	0.88 (0.68–1.13)	0.79 (0.59–1.07)	0.57 (0.40–0.82)	3.40 (3.00–3.85)	1.51^*^ **(1.29–1.76)**	1.32 (1.19–1.47)	1.00 (0.88–1.14)
Non-married	1.67 (1.50–1.86)	0.81 (0.71–0.93)	2.06 (1.76–2.41)	0.97 (0.80–1.18)	2.06 (1.57–2.72)	1.03 (0.75–1.42)	1.13 (0.80–1.60)	0.26^*^ **(0.17–0.39)**	4.39 (3.79–5.08)	1.30 (1.08–1.56)	2.07 (1.83–2.34)	1.08 (0.93–1.25)
Living with partner	1.64 (1.38–1.94)	0.98 (0.81–1.18)	2.32 (1.84–2.92)	1.13 (0.87–1.47)	1.64 (1.04–2.59)	0.94 (0.58–1.54)	2.22 (1.48–3.32)	0.55 (0.34–0.89)	2.88 (2.26–3.68)	1.09 (0.82–1.44)	2.02 (1.67–2.45)	1.12 (0.91–1.39)
Homeownership												
Owner	1.00	1.00	1.00	1.00	1.00	1.00	1.00	1.00	1.00	1.00	1.00	1.00
Renter	2.11 (1.95–2.29)	1.33^*^ **(1.20–1.48)**	2.38 (2.10–2.69)	1.49^*^ **(1.27–1.75)**	1.78 (1.42–2.23)	0.86 (0.65–1.13)	3.82 (3.01–4.86)	1.72 (1.25–2.36)	5.04 (4.50–5.66)	1.70^*^ **(1.46–1.98)**	2.08 (1.89–2.30)	1.23 (1.09–1.40)

*Adj* adjusted, *IHS* Indian health service, *NH* non-Hispanic, *NHPI* Native Hawaiian, Pacific Islander, *Prev* prevalence, *Unadj.* unadjusted

^1^Delayed healthcare due to affordability reasons includes need of out-of-pocket payment, too high deductibles and cannot afford co-pay

^*^P < 0.000397 (Bonferroni-corrected significance level) for adjusted analyses

### Age and delayed healthcare

**Fig. 2:**
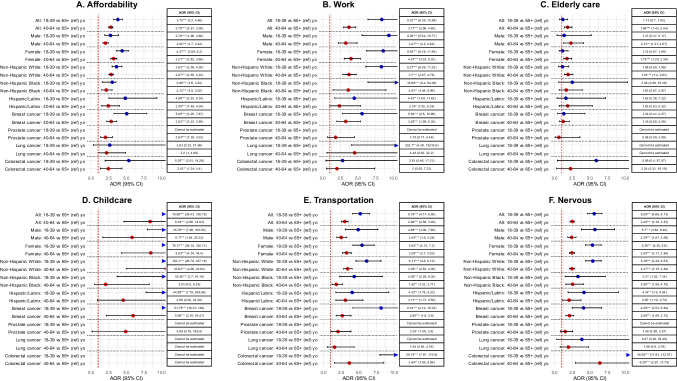
Association between age group (reference group = 65 + years old) and various barriers underlying delayed healthcare across select strata. AOR, adjusted odds ratio; CI, confidence interval. *P < 0.05, **P < 0.01, ***P < 0.001

Younger age was consistently associated with higher odds of delayed healthcare for all evaluated reasons (Fig. 2, Table 3, eTable S1). The youngest group (i.e., 18–39 years, young adults) had consistently higher odds of delayed healthcare (Fig. 2, eTables S2-S6). Younger colorectal cancer survivors were more prone to delaying healthcare due to nervousness about seeing a provider (Fig. 2, eTable S6).

When stratified by age group, not having health insurance (reference = private insurance) was more strongly associated with delayed healthcare due to affordability and transportation barriers among young adults with cancer than older survivors (eTable S2). Additionally, NHB young adults were more likely to report delayed healthcare due to work-related barriers than their NHW counterparts, but this difference was not observed in older age groups (40–64 and 65 + years old) (eTable S2). However, a few associations were stronger in older ages: delayed healthcare was associated with work-related barriers among Hispanic/Latinx (reference = NHW) survivors aged 65 + years, childcare barriers among home renters (reference = owners) aged 40–64 years, transportation barriers among unmarried/separated (reference = married) survivors aged 65 + years, and affordability barriers among survivors with < $25,000 annual household income (reference = $50,000 to < $100,000) in ages 40 + years (eTable S2).

### Biological sex and delayed healthcare

**Fig. 3:**
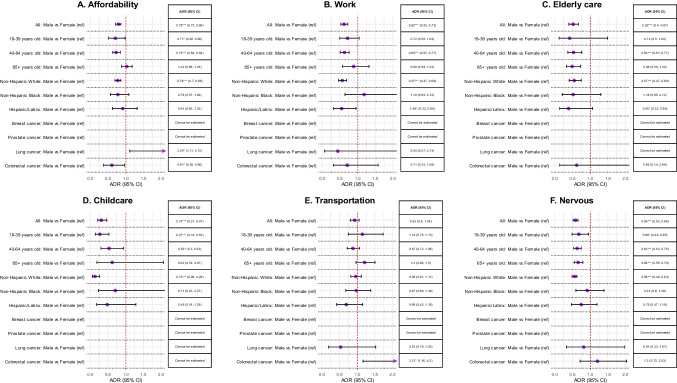
Association between male biological sex (reference group = female) and various barriers underlying delayed healthcare across select strata. AOR, adjusted odds ratio; CI, confidence interval. *P < 0.05, **P < 0.01, ***P < 0.001

Across all strata, male cancer survivors had consistently lower adjusted odds of delayed healthcare for all investigated reasons than females or odds were similar, with 2 exceptions (Fig. 3, Table 3, eTable S1). Compared to their female counterparts, males with lung cancer had 2.29-times (95% CI = 1.11–4.72) higher odds of reporting affordability-related delayed healthcare and males with colorectal cancer had 2.21-times (95% CI = 1.16–4.20) higher odds of transportation-related delayed healthcare (Fig. 3, eTables S5-S6).

In analyses stratified by biological sex, female cancer survivors who rented their homes were more likely to delay healthcare due to affordability barriers and nervousness, compared to female homeowners. This difference was not as pronounced within male cancer survivors (eTable S3). Additionally, a younger age was a stronger predictor of affordability-related delayed healthcare among females compared to males (eTable S3). In contrast, some characteristics were more strongly associated with delayed healthcare among male cancer survivors, including work-related delays among NHB individuals (reference = NHW), childcare-related delays among non-Hispanic Asian and/or Native Hawaiian or Pacific Islander (NHA/NHPI) individuals (reference = NHW) and home renters (reference = owners), and nervousness-related delays among unmarried/separated individuals (reference = married) (eTable S3).

### Race/ethnicity and delayed healthcare

**Fig. 4:**
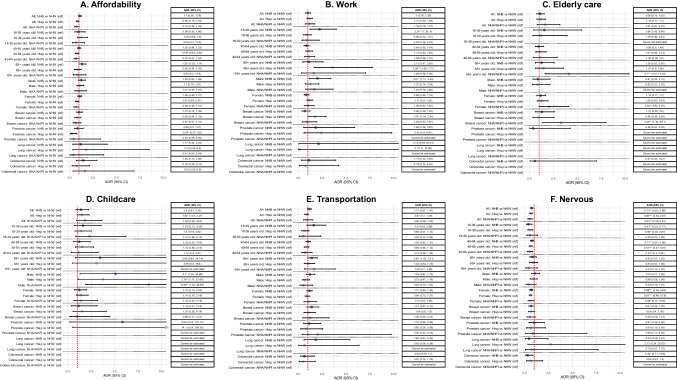
Association between minority racial/ethnic groups (reference group = NHW) and various barriers underlying delayed healthcare across select strata. AOR, adjusted odds ratio; CI, confidence interval; Hisp, Hispanic/Latinx; NHA, non-Hispanic Asian; NHB, non-Hispanic Black; Native Hawaiian/Pacific Islander, NHPI. *P < 0.05, **P < 0.01, ***P < 0.001

Compared to NHW, NHB and Hispanic/Latinx individuals had higher odds of delaying healthcare in unadjusted analyses, but these associations were largely attenuated in adjusted models with Bonferroni correction. The only association that persisted in adjusted models was lower odds of delaying healthcare due to nervousness to see a provider among Hispanic/Latinx than NHW cancer survivors (OR = 0.66, 95% CI = 0.53–0.81) (Fig. 4, Table 3, eTable S1). This association was consistently observed in ages < 65 years and among females (Fig. 4, eTables S2 and S3).

While race/ethnicity was not associated with other delayed healthcare reasons in full cohort analyses, some significant associations were observed in stratified analyses (Fig. 4). For example, compared to their NHW counterparts, NHB young adults and NHB male cancer survivors were more likely to report delayed healthcare due to work (Fig. 4, eTables S2 and S3). NHB cancer survivors also had higher odds of delayed healthcare due to affordability and transportation barriers in older age groups compared to NHW survivors (Fig. 4, eTable S2). Hispanic cancer survivors were more likely to delay healthcare than NHW survivors due to work-related barriers among older adults and breast cancer survivors (Fig. 4, eTables S2 and S5). NHA/NHPI individuals were more likely than NHW to delay healthcare due to elderly care among older adults, females, and breast cancer survivors (Fig. 4, eTables S2, S3 and S5).

Compared to females, male cancer survivors were generally less likely to delay healthcare due to work, childcare and nervousness to see a provider, with much lower odds among NHW than NHB (all above 3 reasons) and Hispanic (childcare) cancer survivors (eTable S4). In contrast, the association between being uninsured or covered by Medicaid (reference = private insurance), unemployed (reference = employed), or renting (reference = owning a home) and delayed healthcare due to affordability, childcare, transportation, and work-related barriers were significantly greater among NHW than among NHB cancer survivors (eTable S4).

### Other sociodemographic characteristics and delayed healthcare

**Fig. 5:**
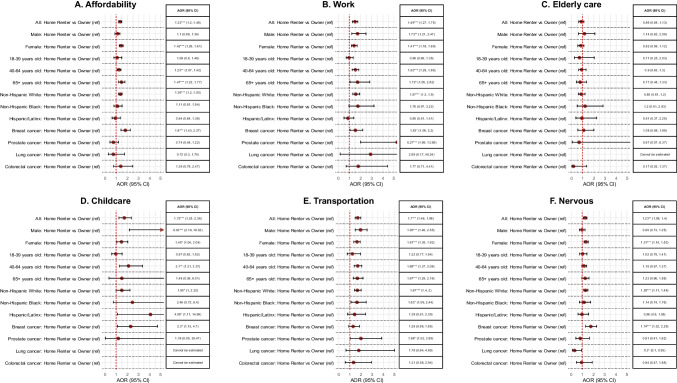
Association between home renters (reference group = homeowners) and various barriers underlying delayed healthcare across select strata. AOR, adjusted odds ratio; CI, confidence interval. *P < 0.05, **P < 0.01, ***P < 0.001

Home renters were at higher odds of reporting delayed healthcare due to affordability, work, and transportation barriers than homeowners even after adjustment for education attainment, household income, employment, and health insurance status and Bonferroni correction (Fig. 5, Table 3, eTable S1). Findings were generally consistent in analyses stratified by sociodemographic characteristics (Fig. 5, eTables S2-S6).

Other notable characteristics that were associated with higher odds of delayed healthcare after Bonferroni correction included elderly care, childcare, and transportation barriers among Medicaid enrollees (reference = private insurance); affordability and transportation barriers among individuals with annual household incomes of < $50,000 (reference = $50,000 to < $100,000); transportation barriers among non-workers (reference = employed); and transportation barriers among Medicare/Dual eligibility enrollees and uninsured survivors (reference = private insurance, reason = affordability), and unmarried/separated survivors (reference = married, reason = transportation) (Table 3, eTable S1).

## Discussion

In this large, nationwide study of cancer survivors in the U.S., we found considerable differences in delayed healthcare and its reasons by several predisposing and enabling factors within the Andersen’s Behavioral Model of Health Care Use [20]. Our Bonferroni-corrected adjusted analysis found that age and sex were associated with all six evaluated reasons for delayed healthcare, and insurance, income, employment, and homeownership were associated with some reasons. There were variations in the strengths of these associations across combinations of sociodemographic characteristics, indicating intersectionality. For example, NHB survivors were more likely than NHW to report delayed healthcare due to unaffordability among the young adults, but such difference was not found in older age groups. This information can provide additional insight into factors contributing to disparities in access to care and opportunities for reducing these disparities, improving survival and survivorship outcomes, and enhancing quality of life across evaluated populations. Herein, we discuss barriers to care among young adult cancer survivors and by biological sex and race/ethnicity, and the role of homeownership in delayed healthcare, as these domains represent key areas of unmet clinical and research needs in cancer survivorship.

### Barriers to healthcare among young adult cancer survivors

As with previous studies, we found that younger cancer survivors, especially young adults, generally experienced more barriers to healthcare access as they were more likely to have limited health insurance coverage and wealth, and thus, more likely to experience financial toxicity compared to older adults [32–37]. Delayed healthcare due to work-related factors can stem from prioritizing early career development, ensuring employment-related health access for self and family, while coping with productivity-reducing effects of cancer, ongoing survivorship care needs, and challenges with meeting expectations amidst fear of discrimination based on medical history [38–40]. The significant gap in healthcare access between privately insured and uninsured young adults in this study underscored the importance of insurance enrollment as a modifiable factor. Current health systems and policies remain inadequate to facilitate access to care for younger cancer survivors with these unique needs [41–44]. The demand for cancer care in younger adults is likely to grow given the increasing incidence of several cancer types in this population, such as colorectal and endometrial cancer [45, 46]. Crucially, young age is a stronger predictor of nervousness before a medical appointment among colorectal cancer compared to other cancers in our study, suggesting significant unmet psychosocial needs in this population. Increasing insurance coverage, including the adoption of Medicaid expansion in all states, can alleviate access-to-care barriers in young adults [47]. Efforts may also be placed on facilitating paid sick leaves, hybrid education and work arrangements to allow flexibility of time for younger cancer survivors to seek the necessary care they require despite their busy schedules [11, 48–50]. Other interventions that may increase healthcare engagement include setting up childcare facilities within the vicinity of cancer centers and developing specialized clinics to address and accommodate the unique needs of younger cancer patients with small children [51, 52].

### Barriers to healthcare by biological sex

In this study, female cancer survivors were more likely than males to experience delays in healthcare for several reasons, including affordability, work responsibilities, elderly care, childcare, and nervousness about seeing a provider. Other studies have similarly reported higher financial toxicity among female than male cancer survivors [53–55]. Increased nervousness with seeing healthcare providers among females may in part stem from negative experiences with previous healthcare encounters, such as anxiety about cancer recurrence, fear of receiving bad news, and challenges related to patient-provider communication [56–58]. These challenges increase the risk of follow-up loss with healthcare providers and underscore the need for systemic changes to reduce disparities by sex, including actions proposed by the *Lancet* Commission on women, power and cancer [57], such as co-creating accessible, responsive and equitable health systems, and ensuring fair economic support for caregivers [59].

In this study, however, males with lung or colorectal cancer were more likely to experience delayed healthcare because of affordability and transportation barriers, respectively. This observation could in part be explained by the lower socioeconomic status among male lung cancer survivors compared to females [60]. It is unclear why transportation barriers were observed more frequently among male colorectal cancer survivors compared to females [61–63]. More research is required to understand and mitigate sex-associated reasons for delayed healthcare among cancer survivors.

### Barriers to care by race/ethnicity

By race/ethnicity, higher impact of work-associated barriers on NHB young adults and older Hispanic/Latinx survivors suggest that these groups may experience limited access to flexible work arrangements to navigate cancer treatment and survivorship. Elderly care commitments weighed more heavily on older, female, and breast cancer survivors in the NHA/NHPI population, a finding consistent with existing literature and underscoring the critical need for targeted interventions for cancer survivors who are also caregivers [64–66]. Male cancer survivors from minoritized racial/ethnic groups were more likely than NHW males to delay healthcare due to childcare responsibilities. Nevertheless, NHW survivors were more prone to delaying healthcare due to provider-related nervousness than minoritized racial/ethnic groups. The impact of lower socioeconomic status (e.g., being uninsured, covered by Medicaid, unemployed, or renting) on healthcare delays was also significantly greater among NHW survivors, revealing yet another frequently unaddressed interaction of race/ethnicity and socioeconomic status within healthcare access.

### Role of homeownership in delayed healthcare

Homeownership can be both a predisposing and enabling factor for delayed healthcare (Fig. 1). Prior evidence has linked housing insecurity to poorer healthcare access, and this study contributes to this literature by demonstrating that homeownership status is independently associated with delayed healthcare even after adjusting for socioeconomic characteristics and multiple testing [67]. A previous study reported that newly diagnosed cancer survivors with housing instability concerns were at two-times higher mortality risk after adjusting for other social risk factors [68]. Owning a house provides a stable place to live in and indicates the availability of wealth to liquidate or mortgage in case of emergent expenditure [69]. On the other hand, renters may face financial constraints due to rising rental costs in many parts of the U.S., which may lead to a prioritization of work and immediate financial needs over investing in cancer care [70] Transitioning between accommodations could also lead to losses to follow-up care among renters [70]. In this study, the impact of not owning a house on delayed healthcare was significantly greater in cancer survivors aged 40 and over, indicating considerable unmet needs among less wealthy individuals in this group. Certain interventions may reduce housing insecurity and its adverse effects on health. At the providers’ level, routine screening for housing insecurity is recommended to ensure timely referrals to community resources to address cancer survivors’ needs. This entails strong connections between cancer centers with community-based organizations, where newly diagnosed patients with housing insecurity could be referred to financial navigation, temporary accommodations, and rental assistance, thereby reducing the risk of delay to treatment [67, 71]. Enabling telemedicine services and interoperability of healthcare institutions may also be helpful to cancer survivors living in neighborhoods with limited accessibility to healthcare facilities [72, 73].

### Intersectionality and barriers to care

Intersectionality refers to the way in which multiple social identities, such as age, sex, and race/ethnicity, simultaneously shape an individual’s experience (in this study, healthcare barriers), such that the effect of one characteristic may differ depending on the presence or level of another. Of note, research on intersectionality across the cancer care continuum has been limited [74, 75]. Although we found variations in reasons for delayed healthcare among cancer survivors after stratification of results by several social identities, the stratified analyses were a preliminary approximation instead of a purpose-built intersectionality-informed analysis across multiple social identities. Future research should administer intersectionality-focused methods, such as the multilevel analysis of individual heterogeneity and discriminatory accuracy (MAIHDA) [76, 77]. Qualitative and community-based participatory approaches could further illuminate the structural and social mechanisms (e.g. stigma, discrimination, and power dynamics) underlying observed disparities.

### Strengths and limitations

A key strength of this study is its large sample size, which enables robust, comprehensive analyses. However, a cross-sectional design is a limitation of this study, as reverse causality may be a concern with any observed associations. Due to the lack of data, we could not take into account distance to care and prior healthcare experiences, which could have provided further insight, especially when examining the effects of transportation barriers and nervousness on delayed healthcare. The use of self-report data increases the risk of recall bias that could lead to over- or under-estimation of effect sizes, indicating the need for further research based on validated data, such as EHR-derived guideline-concordant endpoints. Racial-ethnic groups other than those included in this study, minoritized sexual-gender groups, and unhoused populations are under-represented in the data that were used in this study [26, 78]. The predominance of non-Hispanic White participants may lead to an inaccurate estimation of underlying racial/ethnic disparities, specifically where null findings in the full-cohort adjusted models should be interpreted with caution given the limited statistical power for smaller racial/ethnic groups. As such, reasons for delayed healthcare in these under-represented populations should be further evaluated.

## Conclusion

Delayed healthcare among cancer survivors varies significantly by age, sex, insurance, income, employment, and homeownership. Findings highlight the need for tailored interventions to effectively address the unique social needs of each cancer survivor, ultimately improving healthcare access for all. Expanding health insurance coverage, such as nationwide Medicaid expansion, offering financial navigation services, and supporting paid sick leave and flexible work arrangements may help reduce barriers across many sociodemographic groups. Future research should evaluate these strategies through an intersectional lens to address the complex challenges faced by diverse cancer survivor populations.

## Supplementary Information

Below is the link to the electronic supplementary material.

ESM 1 (DOCX 338 KB)

## Data Availability

The All of Us Research Hub employs a tiered data access model with three levels (Public Tier, Registered Tier, and Controlled Tier). The Controlled Tier includes data from electronic health records (EHRs), wearables, surveys, and physical measurements collected at participant enrollment, as well as genomic data from whole-genome sequencing and genotyping arrays. Unlike the Registered Tier, restricted demographic data fields from EHRs and surveys, and unshifted event dates are made available in the Controlled Tier. Currently, the Controlled Tier data are accessible to researchers affiliated with academic institutions, non-profit organizations, and health care institutions (both non-profit and for-profit), with plans to extend access to additional groups, including industry-affiliated researchers. To access the Controlled Tier, researchers must complete the All of Us Researcher Workbench access process, which includes identity verification and specific training on human participant research (https://www.researchallofus.org/register/). Researchers may create a workspace at any time to conduct studies, provided they comply with Data Use Policies and self-declare their research purpose. This information is publicly available on the All of Us Research Projects Directory at https://allofus.nih.gov/protecting-data-and-privacy/research-projects-all-us-data.
