# Supplementary material for: Sociodemographic disparities and reasons for delayed healthcare among U.S. cancer survivors: an All of Us study
Source: Support Care Cancer. 2026 Jun 16;34(7):663. doi: 10.1007/s00520-026-10880-y (PMC13272612; doi:10.1007/s00520-026-10880-y)
Supplement: Supplementary file 1 — (DOCX 338 KB) [file 520_2026_10880_MOESM1_ESM.docx]

**Supplementary Online Content**

Sociodemographic Disparities and Reasons for Delayed Healthcare Among U.S. Cancer Survivors: An All of Us Study (2026 Ng et al.)

**eFigure S1.** Cohort selection

**eTable S1.** Summary of significant sociodemographic associations and odds ratios for delayed healthcare reasons in multivariate analyses after Bonferroni correction (P<0.000397)

**eTable S2.** Factors associated with various reasons for delayed healthcare, stratified by age at survey

**eTable S3.** Factors associated with various reasons for delayed healthcare, stratified by biological sex

**eTable S4.** Factors associated with various reasons for delayed healthcare, stratified by racial/ethnic background

**eTable S5.** Factors associated with various reasons for delayed healthcare (affordability, work and elderly care), stratified by cancer type

**eTable S6.** Factors associated with various reasons for delayed healthcare (childcare, transportation and nervous), stratified by cancer type

**eFigure S1.** Cohort selection


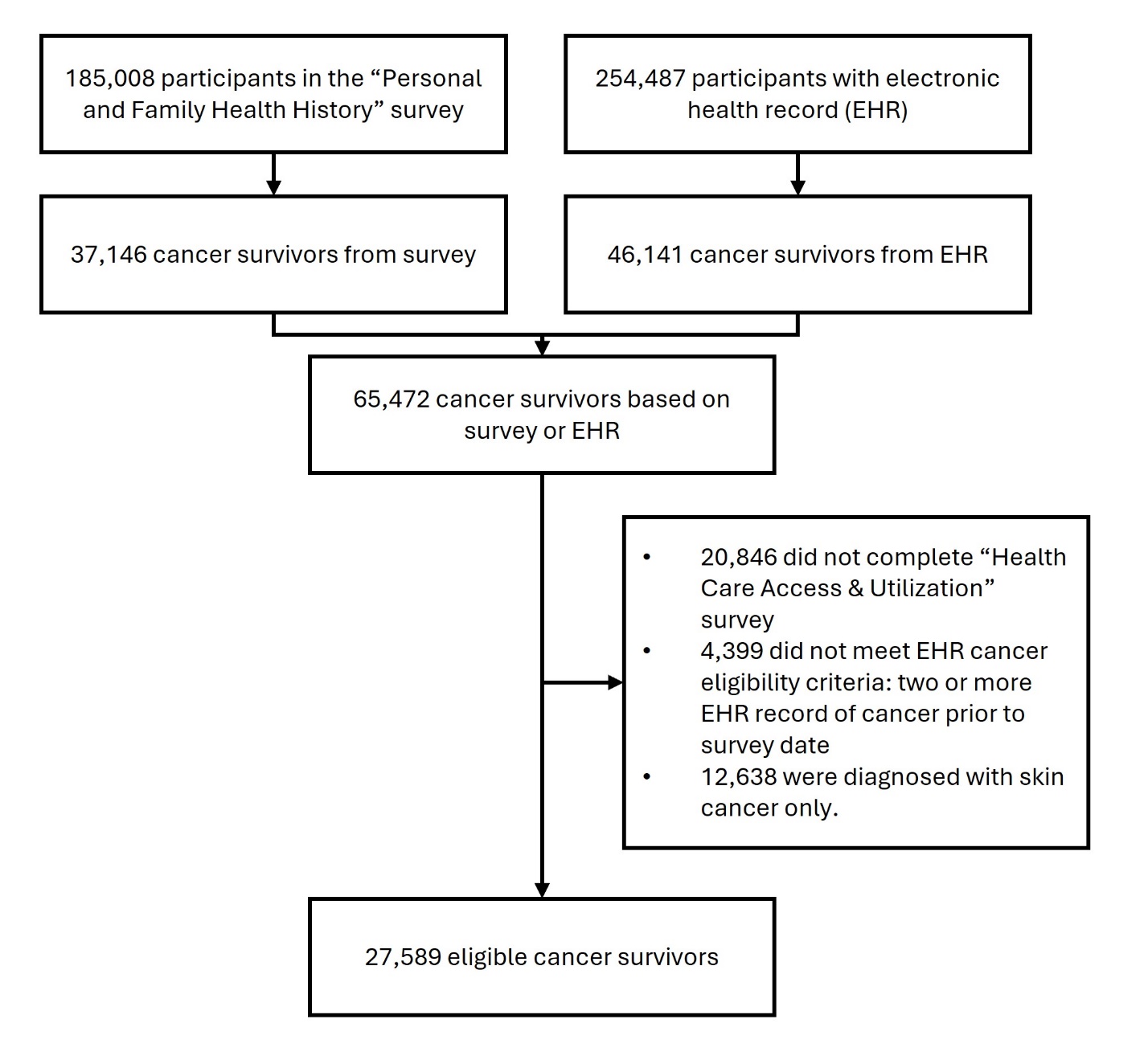


**eTable S1.** Summary of significant sociodemographic associations and odds ratios for delayed healthcare reasons in multivariate analyses after Bonferroni correction (P<0.000397)

| **Characteristics** | **Reasons for delayed healthcare** | **Significant sociodemographic associations (Bonferroni-corrected P<0.000397) and ORs^1^** |
| --- | --- | --- |
| Age at survey | Affordability | - 18-39 years old vs 65+ years old (ref): OR=3.79 - 40-64 years old vs 65+ years old (ref): OR=2.78 |
|  | Work | - 18-39 years old vs 65+ years old (ref): OR=8.33 - 40-64 years old vs 65+ years old (ref): OR=3.71 |
|  | Elderly care | - 40-64 years old vs 65+ years old (ref): OR=1.86 |
|  | Childcare | - 18-39 years old vs 65+ years old (ref): OR=70.89 - 40-64 years old vs 65+ years old (ref): OR=8.34 |
|  | Transportation | - 18-39 years old vs 65+ years old (ref): OR=5.19 - 40-64 years old vs 65+ years old (ref): OR=2.99 |
|  | Nervousness | - 18-39 years old vs 65+ years old (ref): OR=5.59 - 40-64 years old vs 65+ years old (ref): OR=2.48 |
| Biological sex | Affordability | - Male vs Female (ref): OR=0.79 |
|  | Work | - Male vs Female (ref): OR=0.62 |
|  | Elderly care | - Male vs Female (ref): OR=0.52 |
|  | Childcare | - Male vs Female (ref): OR=0.31 |
|  | Nervousness | - Male vs Female (ref): OR=0.59 |
| Race/ethnicity | Nervousness | - Hispanic vs Non-Hispanic White (ref): OR=0.66 |
| Education attainment |  |  |
| Annual income | Affordability | - <25k vs 50-100k (ref): OR=1.62 - 25-50k vs 50-100k (ref): OR=1.62 - 100-200k vs 50-100k (ref): OR=0.62 - ≥200k vs 50-100k (ref): OR=0.35 |
|  | Transportation | - <25k vs 50-100k (ref): OR=2.78 - 25-50k vs 50-100k (ref): OR=1.59 |
|  | Nervousness | - <25k vs 50-100k (ref): OR=1.67 |
| Employment | Affordability | - Not working vs Employed (ref): OR=0.76 |
|  | Work | - Not working vs Employed (ref): OR=0.16 |
|  | Transportation | - Not working vs Employed (ref): OR=1.86 |
| Health insurance | Affordability | - Medicaid vs Private (ref): OR=0.54 - Uninsured vs Private (ref): OR=2.07 |
|  | Work | - Medicare/Dual eligibility vs Private (ref): OR=0.53 |
|  | Elderly care | - Medicaid vs Private (ref): OR=1.94 |
|  | Childcare | - Medicaid vs Private (ref): OR=2.38 |
|  | Transportation | - Medicare/Dual eligibility vs Private (ref): OR=1.56 - Medicaid vs Private (ref): OR=1.49 |
| Marital status | Childcare | - Non-married vs Married (ref): OR=0.26 |
|  | Transportation | - Divorced/Separated/Widowed vs Married (ref): OR=1.51 |
| Homeownership | Affordability | - Renter vs Owner (ref): OR=1.33 |
|  | Work | - Renter vs Owner (ref): OR=1.49 |
|  | Transportation | - Renter vs Owner (ref): OR=1.70 |

Abbreviations: OR, odds ratios; ref, reference group.

^1^ Full results from analysis are reported in **Table 3**.

**eTable S2.** Factors associated with various reasons for delayed healthcare, stratified by age at survey

|  | **Affordability^1^** | | | **Work** | | | **Elderly care** | | | **Childcare** | | | **Transportation** | | | **Nervous** | | |
| --- | --- | --- | --- | --- | --- | --- | --- | --- | --- | --- | --- | --- | --- | --- | --- | --- | --- | --- |
|  | **Adj. OR (95% CI)** | | | **Adj. OR (95% CI)** | | | **Adj. OR (95% CI)** | | | **Adj. OR (95% CI)** | | | **Adj. OR (95% CI)** | | | **Adj. OR (95% CI)** | | |
|  | **18-39** | **40-64** | **65+** | **18-39** | **40-64** | **65+** | **18-39** | **40-64** | **65+** | **18-39** | **40-64** | **65+** | **18-39** | **40-64** | **65+** | **18-39** | **40-64** | **65+** |
| **Biological sex** |  |  |  |  |  |  |  |  |  |  |  |  |  |  |  |  |  |  |
| Female | 1.0 | 1.0 | 1.0 | 1.0 | 1.0 | 1.0 | 1.0 | 1.0 | 1.0 | 1.0 | 1.0 | 1.0 | 1.0 | 1.0 | 1.0 | 1.0 | 1.0 | 1.0 |
| Male | **0.71*^†^**  **(0.52-0.98)** | **0.73***^‡^**  **(0.64-0.84)** | **1.02^†‡^**  **(0.88-1.18)** | 0.72  (0.50-1.04) | 0.63***  (0.51-0.77) | 0.88  (0.58-1.32) | 0.42  (0.12-1.49) | 0.53**  (0.37-0.77) | 0.49***  (0.33-0.73) | 0.27***  (0.14-0.53) | 0.53*  (0.30-0.93) | 0.62  (0.19-2.07) | 1.14  (0.75-1.73) | **0.87^‡^**  **(0.72-1.06)** | **1.20^‡^**  **(0.96-1.50)** | 0.68*  (0.49-0.95) | 0.63***  (0.54-0.75) | 0.66***  (0.55-0.79) |
| **Race/ethnicity** |  |  |  |  |  |  |  |  |  |  |  |  |  |  |  |  |  |  |
| NH-White | 1.0 | 1.0 | 1.0 | 1.0 | 1.0 | 1.0 | 1.0 | 1.0 | 1.0 | 1.0 | 1.0 | 1.0 | 1.0 | 1.0 | 1.0 | 1.0 | 1.0 | 1.0 |
| NH-Black | 0.96  (0.56-1.66) | 1.02  (0.84-1.23) | 1.33*  (1.02-1.74) | **2.24**^§^**  **(1.26-4.00)** | **0.84^§^**  **(0.62-1.14)** | 1.11  (0.47-2.62) | 1.54  (0.43-5.46) | 0.95  (0.60-1.49) | 1.26  (0.62-2.56) | 1.61  (0.73-3.56) | 1.22  (0.62-2.42) | 3.46  (0.81-14.74) | 1.61  (0.90-2.88) | 1.03  (0.82-1.31) | 1.43*  (1.02-2.01) | 0.41**  (0.22-0.77) | 0.77*  (0.61-0.98) | 0.83  (0.57-1.22) |
| Hispanic | **1.26^§^**  **(0.84-1.90)** | **0.79*^§^**  **(0.64-0.98)** | 0.82  (0.54-1.25) | **0.85^†^**  **(0.53-1.37)** | **1.11^‡^**  **(0.83-1.49)** | **3.59**^†‡^**  **(1.66-7.77)** | 1.19  (0.36-3.94) | 1.07  (0.66-1.73) | 1.47  (0.60-3.56) | 1.40  (0.78-2.52) | 1.51  (0.83-2.75) | 3.05  (0.50-18.40) | 0.65  (0.37-1.14) | 0.85  (0.65-1.12) | 1.04  (0.63-1.73) | 0.56*  (0.35-0.87) | 0.61***  (0.47-0.81) | 0.84  (0.51-1.38) |
| NH-Asian/NHPI | 0.69  (0.30-1.59) | 0.70  (0.47-1.04) | 0.84  (0.40-1.74) | 0.92  (0.39-2.19) | 1.08  (0.68-1.72) | 1.65  (0.38-7.05) | - | 1.05  (0.38-2.90) | 4.10**  (1.62-10.38) | 2.31  (0.86-6.19) | 1.14  (0.40-3.27) | - | 0.53  (0.12-2.36) | 0.74  (0.38-1.45) | 1.65  (0.70-3.86) | 0.56  (0.23-1.38) | 0.51*  (0.30-0.85) | 0.42  (0.13-1.32) |
| **Education attainment** |  |  |  |  |  |  |  |  |  |  |  |  |  |  |  |  |  |  |
| High school diploma or less | **1.72*^§^**  **(1.08-2.75)** | **0.81*^§‡^**  **(0.66-0.98)** | **1.18^‡^**  **(0.92-1.51)** | 1.72*  (1.01-2.95) | 0.96  (0.71-1.30) | 1.29  (0.62-2.71) | 1.18  (0.24-5.80) | 1.26  (0.77-2.04) | 0.68  (0.36-1.29) | 0.87  (0.43-1.73) | 0.44*  (0.22-0.87) | 1.94  (0.25-14.90) | 1.58  (0.84-2.97) | 1.43*  (1.07-1.91) | 1.21  (0.84-1.74) | 0.98  (0.61-1.58) | 1.05  (0.84-1.33) | 1.30  (0.97-1.76) |
| Some college | 1.17  (0.80-1.72) | 1.22**  (1.05-1.42) | 1.12  (0.92-1.35) | 1.67*  (1.10-2.56) | 1.19  (0.96-1.48) | 0.94  (0.55-1.62) | 1.03  (0.23-4.59) | 1.19  (0.77-1.81) | 0.78  (0.49-1.22) | 1.15  (0.64-2.01) | 0.58*  (0.34-1.00) | 0.91  (0.12-7.14) | 1.42  (0.79-2.53) | 1.24  (0.95-1.62) | 1.36*  (1.02-1.82) | 1.17  (0.79-1.72) | **1.11^‡^**  **(0.92-1.33)** | **0.81^‡^**  **(0.63-1.04)** |
| Bachelor | 1.35  (0.96-1.90) | 1.00  (0.86-1.16) | 1.19  (0.99-1.42) | **1.59*^§^**  **(1.10-2.32)** | **1.01^§^**  **(0.83-1.23)** | 0.91  (0.56-1.47) | 1.62  (0.38-6.85) | **1.20^‡^**  **(0.79-1.83)** | **0.60*^‡^**  **(0.37-0.95)** | 0.98  (0.58-1.65) | **0.70^‡^**  **(0.43-1.15)** | **4.61^‡^**  **(0.95-22.23)** | 1.18  (0.66-2.11) | 1.24  (0.95-1.63) | 1.06  (0.79-1.43) | 1.01  (0.70-1.44) | 1.07  (0.90-1.27) | 1.05  (0.85-1.30) |
| Master or more | 1.0 | 1.0 | 1.0 | 1.0 | 1.0 | 1.0 | 1.0 | 1.0 | 1.0 | 1.0 | 1.0 | 1.0 | 1.0 | 1.0 | 1.0 | 1.0 | 1.0 | 1.0 |
| **Annual household income** |  |  |  |  |  |  |  |  |  |  |  |  |  |  |  |  |  |  |
| Less than $25,000 | **0.90^†^**  **(0.57-1.40)** | **1.47***^‡^**  **(1.19-1.81)** | **2.29***^†‡^**  **(1.77-2.95)** | 0.95  (0.56-1.61) | 1.31  (0.93-1.86) | 1.71  (0.77-3.81) | 2.33  (0.61-8.94) | 1.51  (0.91-2.48) | 1.84  (0.97-3.48) | 0.75  (0.38-1.45) | 0.72  (0.36-1.44) | 1.94  (0.36-10.51) | 2.47**  (1.43-4.27) | 2.50***  (1.86-3.36) | 2.98***  (2.09-4.24) | 2.02**  (1.29-3.16) | 1.46**  (1.14-1.88) | 1.65**  (1.18-2.29) |
| $25,000 - $49,999 | 1.32  (0.92-1.90) | 1.62***  (1.36-1.92) | 1.76***  (1.44-2.14) | 1.24  (0.83-1.85) | 1.38*  (1.07-1.78) | 1.02  (0.54-1.95) | 1.10  (0.28-4.34) | 1.54  (0.97-2.43) | 1.58  (0.98-2.55) | **1.47^§^**  **(0.85-2.54)** | **0.48*^§^**  **(0.23-0.98)** | 0.38  (0.04-3.59) | 1.11  (0.64-1.93) | 1.67***  (1.25-2.23) | 1.58**  (1.14-2.20) | 1.54*  (1.05-2.27) | 1.05  (0.84-1.32) | 1.02  (0.78-1.35) |
| $50,000 - $99,999 | 1.0 | 1.0 | 1.0 | 1.0 | 1.0 | 1.0 | 1.0 | 1.0 | 1.0 | 1.0 | 1.0 | 1.0 | 1.0 | 1.0 | 1.0 | 1.0 | 1.0 | 1.0 |
| $100,000 - $199,999 | 0.50***  (0.35-0.73) | 0.57***  (0.49-0.67) | 0.72**  (0.58-0.89) | 0.71  (0.47-1.07) | 0.83  (0.67-1.03) | 0.84  (0.47-1.48) | 0.82  (0.18-3.64) | 0.61  (0.36-1.02) | 0.48*  (0.27-0.86) | 0.87  (0.50-1.54) | 0.41**  (0.22-0.75) | 1.21  (0.26-5.63) | 0.51  (0.25-1.05) | 0.57**  (0.40-0.82) | 0.92  (0.62-1.37) | 0.93  (0.63-1.37) | 0.88  (0.73-1.07) | 0.92  (0.71-1.18) |
| $200,000 and above | **0.15***^†^**  **(0.06-0.33)** | **0.32***^‡^**  **(0.25-0.40)** | **0.49***^†‡^**  **(0.35-0.69)** | **0.46*^†^**  **(0.23-0.91)** | **0.61***^‡^**  **(0.46-0.82)** | **1.35^†‡^**  **(0.72-2.52)** | - | 0.46*  (0.22-0.96) | 0.19**  (0.06-0.63) | 1.00  (0.44-2.29) | 0.75  (0.39-1.43) | - | 0.82  (0.30-2.25) | 0.64  (0.40-1.01) | 0.59  (0.29-1.19) | 0.55  (0.29-1.07) | 0.77*  (0.60-0.99) | 0.82  (0.57-1.17) |
| **Employment status** |  |  |  |  |  |  |  |  |  |  |  |  |  |  |  |  |  |  |
| Employed | 1.0 | 1.0 | 1.0 | 1.0 | 1.0 | 1.0 | 1.0 | 1.0 | 1.0 | 1.0 | 1.0 | 1.0 | 1.0 | 1.0 | 1.0 | 1.0 | 1.0 | 1.0 |
| Not working | **0.99^†^**  **(0.72-1.37)** | **0.97^‡^**  **(0.85-1.10)** | **0.63***^†‡^**  **(0.53-0.74)** | **0.34***^†^**  **(0.23-0.51)** | **0.21***^‡^**  **(0.17-0.27)** | **0.06***^†‡^**  **(0.03-0.10)** | 1.17  (0.47-2.92) | 1.19  (0.86-1.65) | 0.85  (0.55-1.31) | 1.73*  (1.13-2.64) | 2.05**  (1.32-3.18) | 3.73  (0.47-29.62) | 1.57*  (1.07-2.30) | 2.09***  (1.72-2.55) | 1.67**  (1.22-2.28) | 0.98  (0.72-1.34) | 1.25**  (1.08-1.46) | 1.00  (0.81-1.23) |
| **Health insurance** |  |  |  |  |  |  |  |  |  |  |  |  |  |  |  |  |  |  |
| Private | 1.0 | 1.0 | 1.0 | 1.0 | 1.0 | 1.0 | 1.0 | 1.0 | 1.0 | 1.0 | 1.0 | 1.0 | 1.0 | 1.0 | 1.0 | 1.0 | 1.0 | 1.0 |
| Medicare/Dual eligibility | 0.73  (0.38-1.40) | **0.80*^‡^**  **(0.65-1.00)** | **1.11^‡^**  **(0.95-1.28)** | **0.14**^†^**  **(0.03-0.63)** | 0.40***  (0.24-0.66) | **0.71^†^**  **(0.47-1.09)** | 1.66  (0.25-11.15) | **1.98**^‡^**  **(1.23-3.18)** | **1.02^‡^**  **(0.71-1.47)** | 1.16  (0.42-3.19) | 0.79  (0.34-1.87) | 0.90  (0.28-2.89) | 1.70  (0.84-3.43) | **2.02***^‡^**  **(1.55-2.64)** | **1.17^‡^**  **(0.92-1.48)** | 1.18  (0.61-2.27) | 1.01  (0.78-1.30) | 0.89  (0.75-1.07) |
| Medicaid | **0.33***^§^**  **(0.22-0.51)** | **0.56***^§^**  **(0.45-0.69)** | 0.58  (0.31-1.09) | 0.67  (0.43-1.06) | 0.58**  (0.41-0.81) | - | 3.96*  (1.14-13.78) | 2.15***  (1.37-3.38) | 0.47  (0.06-3.56) | 2.35**  (1.36-4.06) | 1.73  (0.90-3.34) | - | 2.13**  (1.32-3.43) | 1.57**  (1.20-2.04) | 1.10  (0.60-2.04) | 1.13  (0.76-1.68) | 1.05  (0.83-1.34) | 0.61  (0.28-1.31) |
| Uninsured (including IHS only, single service plans) | 2.65**  (1.25-5.64) | **2.20***^‡^**  **(1.58-3.02)** | **0.92^‡^**  **(0.43-1.98)** | 0.46  (0.18-1.22) | 0.79  (0.47-1.34) | 0.65  (0.07-5.68) | 8.14*  (1.52-43.52) | 1.27  (0.53-3.08) | 3.33  (0.96-11.50) | 2.97*  (1.12-7.92) | 1.94  (0.72-5.25) | - | **3.81**^§†^**  **(1.71-8.50)** | **1.48^§^**  **(0.94-2.33)** | **0.57^†^**  **(0.17-1.89)** | 2.00  (0.94-4.27) | 0.86  (0.55-1.35) | 1.29  (0.54-3.09) |
| **Marital status** |  |  |  |  |  |  |  |  |  |  |  |  |  |  |  |  |  |  |
| Married | 1.0 | 1.0 | 1.0 | 1.0 | 1.0 | 1.0 | 1.0 | 1.0 | 1.0 | 1.0 | 1.0 | 1.0 | 1.0 | 1.0 | 1.0 | 1.0 | 1.0 | 1.0 |
| Divorced/Separated/Widowed | 1.27  (0.81-2.00) | 0.91  (0.79-1.06) | 1.08  (0.91-1.28) | 1.22  (0.73-2.02) | 1.19  (0.97-1.46) | 1.42  (0.87-2.32) | 0.42  (0.12-1.48) | 0.96  (0.66-1.38) | 0.85  (0.56-1.31) | 0.54*  (0.29-1.00) | 0.57*  (0.34-0.96) | 2.18  (0.62-7.70) | **1.02^†^**  **(0.59-1.78)** | **1.25*^‡^**  **(1.01-1.55)** | **2.56***^†‡^**  **(1.95-3.36)** | 0.91  (0.57-1.44) | 1.09  (0.91-1.30) | 1.14  (0.92-1.41) |
| Non-married | 0.94  (0.68-1.31) | **0.75**^‡^**  **(0.63-0.90)** | **1.05^‡^**  **(0.80-1.36)** | **0.89^†^**  **(0.61-1.28)** | **0.84^‡^**  **(0.65-1.09)** | **2.40**^†‡^**  **(1.30-4.45)** | 0.92  (0.35-2.42) | 1.25  (0.83-1.88) | 0.56  (0.26-1.20) | 0.22***  (0.12-0.38) | 0.42**  (0.22-0.78) | - | **0.80^†^**  **(0.51-1.27)** | **1.10^‡^**  **(0.86-1.41)** | **2.48***^†‡^**  **(1.72-3.58)** | 0.82  (0.58-1.15) | 1.05  (0.85-1.29) | 1.30  (0.95-1.78) |
| Living with partner | 1.12  (0.74-1.70) | 1.05  (0.82-1.35) | 0.78  (0.49-1.24) | 1.06  (0.67-1.68) | 1.13  (0.81-1.59) | 1.99  (0.69-5.69) | - | 1.14  (0.63-2.08) | 1.44  (0.61-3.43) | 0.67  (0.38-1.20) | 0.26*  (0.09-0.77) | - | 0.96  (0.54-1.71) | 0.92  (0.63-1.34) | 1.08  (0.49-2.37) | 1.02  (0.67-1.56) | 1.01  (0.75-1.37) | 1.35  (0.84-2.18) |
| **Homeownership** |  |  |  |  |  |  |  |  |  |  |  |  |  |  |  |  |  |  |
| Owner | 1.0 | 1.0 | 1.0 | 1.0 | 1.0 | 1.0 | 1.0 | 1.0 | 1.0 | 1.0 | 1.0 | 1.0 | 1.0 | 1.0 | 1.0 | 1.0 | 1.0 | 1.0 |
| Renter | 1.09  (0.80-1.49) | 1.23**  (1.07-1.42) | 1.47***  (1.22-1.77) | **0.96^§^**  **(0.69-1.35)** | **1.52***^§^**  **(1.25-1.85)** | 1.72*  (1.05-2.82) | 0.71  (0.25-2.03) | 0.90  (0.63-1.30) | 0.77  (0.45-1.33) | **0.97^§^**  **(0.62-1.52)** | **2.10**^§^**  **(1.31-3.37)** | 1.48  (0.36-6.01) | 1.22  (0.77-1.94) | 1.68***  (1.37-2.06) | 1.67***  (1.29-2.16) | 1.02  (0.75-1.41) | 1.15  (0.97-1.37) | 1.23  (0.96-1.58) |

Abbreviations: Adj., adjusted; CI, confidence interval; IHS, Indian health service; NH, non-Hispanic; NHPI, Native Hawaiian, Pacific Islander; OR, odds ratio.

^1^Delayed healthcare due to affordability reasons includes need of out-of-pocket payment, too high deductibles and cannot afford co-pay.

*P<0.05, **P<0.01, ***P<0.001.

^§^Coefficients are significantly different at 5% significance level (18-39 vs 40-64 years old).

^†^Coefficients are significantly different at 5% significance level (18-39 vs 65+ years old).

^‡^Coefficients are significantly different at 5% significance level (40-64 vs 65+ years old).

(-) Coefficients cannot be estimated.

**eTable S3.** Factors associated with various reasons for delayed healthcare, stratified by biological sex

|  | **Affordability^1^** | | **Work** | | **Elderly care** | | **Childcare** | | **Transportation** | | **Nervous** | |
| --- | --- | --- | --- | --- | --- | --- | --- | --- | --- | --- | --- | --- |
|  | **Adj. OR (95% CI)** | | **Adj. OR (95% CI)** | | **Adj. OR (95% CI)** | | **Adj. OR (95% CI)** | | **Adj. OR (95% CI)** | | **Adj. OR (95% CI)** | |
|  | **Male** | **Female** | **Male** | **Female** | **Male** | **Female** | **Male** | **Female** | **Male** | **Female** | **Male** | **Female** |
| **Age at survey** |  |  |  |  |  |  |  |  |  |  |  |  |
| 18-39 | **2.76***^§^**  **(1.96-3.89)** | **4.37***^§^**  **(3.59-5.30)** | 9.35***  (5.54-15.77) | 8.55***  (6.18-11.84) | 1.37  (0.37-5.17) | 1.12  (0.67-1.89) | 35.78***  (7.98-160.52) | 76.17***  (38.15-152.11) | 4.88***  (3.09-7.69) | 5.45***  (4.13-7.20) | 5.70***  (3.82-8.49) | 5.36***  (4.35-6.60) |
| 40-64 | **2.04***^§^**  **(1.70-2.44)** | **3.21***^§^**  **(2.82-3.66)** | 3.27***  (2.20-4.84) | 4.01***  (3.03-5.32) | 2.31**  (1.31-4.07) | 1.75***  (1.29-2.38) | 5.77**  (1.64-20.32) | 8.43***  (4.34-16.40) | 2.49***  (1.90-3.28) | 3.26***  (2.70-3.93) | 2.39***  (1.87-3.06) | 2.45***  (2.11-2.84) |
| 65+ | 1.0 | 1.0 | 1.0 | 1.0 | 1.0 | 1.0 | 1.0 | 1.0 | 1.0 | 1.0 | 1.0 | 1.0 |
| **Race/ethnicity** |  |  |  |  |  |  |  |  |  |  |  |  |
| NH-White | 1.0 | 1.0 | 1.0 | 1.0 | 1.0 | 1.0 | 1.0 | 1.0 | 1.0 | 1.0 | 1.0 | 1.0 |
| NH-Black | 1.25  (0.93-1.69) | 1.06  (0.89-1.27) | **2.01*^§^**  **(1.17-3.43)** | **0.86^§^**  **(0.65-1.15)** | 0.87  (0.35-2.15) | 1.14  (0.77-1.70) | 5.10*  (1.33-19.66) | 1.21  (0.73-2.02) | 1.23  (0.87-1.75) | 1.17  (0.94-1.45) | 1.05  (0.71-1.56) | 0.68***  (0.54-0.84) |
| Hispanic | 1.10  (0.79-1.51) | 0.80*  (0.65-0.97) | 1.27  (0.74-2.18) | 1.17  (0.90-1.53) | 0.82  (0.31-2.16) | 1.25  (0.81-1.94) | 3.76*  (1.11-12.69) | 1.41  (0.91-2.18) | 0.73  (0.47-1.16) | 0.94  (0.73-1.21) | 0.88  (0.58-1.34) | 0.61***  (0.48-0.78) |
| NH-Asian/NHPI | 1.01  (0.56-1.81) | 0.68  (0.46-1.01) | 1.67  (0.76-3.66) | 1.06  (0.67-1.67) | - | 2.04*  (1.02-4.07) | **8.64**^§^**  **(1.92-38.89)** | **1.42^§^**  **(0.63-3.18)** | 0.54  (0.19-1.52) | 1.07  (0.60-1.93) | 0.43  (0.15-1.19) | 0.58*  (0.37-0.92) |
| **Education attainment** |  |  |  |  |  |  |  |  |  |  |  |  |
| High school diploma or less | 0.95  (0.72-1.25) | 1.01  (0.85-1.20) | 0.80  (0.45-1.44) | 1.20  (0.91-1.57) | 0.84  (0.37-1.90) | 1.02  (0.69-1.51) | 1.16  (0.27-4.89) | 0.66  (0.41-1.08) | 1.17  (0.81-1.70) | 1.55***  (1.20-1.99) | 1.25  (0.88-1.78) | 1.04  (0.85-1.26) |
| Some college | 1.04  (0.84-1.28) | 1.27***  (1.11-1.46) | 1.26  (0.85-1.86) | 1.25*  (1.02-1.53) | 1.09  (0.58-2.06) | 0.95  (0.68-1.32) | 1.12  (0.31-4.03) | 0.86  (0.58-1.27) | 1.02  (0.73-1.41) | 1.50***  (1.20-1.87) | 1.14  (0.86-1.52) | 1.01  (0.87-1.18) |
| Bachelor | 0.99  (0.82-1.20) | 1.14*  (1.00-1.29) | 1.00  (0.70-1.42) | 1.12  (0.93-1.35) | 1.02  (0.54-1.93) | 0.86  (0.62-1.20) | 1.48  (0.49-4.45) | 0.90  (0.63-1.30) | 1.06  (0.77-1.46) | 1.20  (0.95-1.52) | **1.35*^§^**  **(1.05-1.74)** | **0.97^§^**  **(0.84-1.12)** |
| Master or more | 1.0 | 1.0 | 1.0 | 1.0 | 1.0 | 1.0 | 1.0 | 1.0 | 1.0 | 1.0 | 1.0 | 1.0 |
| **Annual household income** |  |  |  |  |  |  |  |  |  |  |  |  |
| Less than $25,000 | 1.81***  (1.36-2.41) | 1.56***  (1.30-1.86) | 1.36  (0.75-2.46) | 1.29  (0.95-1.75) | 1.73  (0.79-3.79) | 1.80**  (1.20-2.71) | 1.11  (0.25-4.96) | 0.81  (0.50-1.30) | 2.98***  (2.03-4.37) | 2.74***  (2.14-3.51) | 1.61*  (1.12-2.31) | 1.70***  (1.39-2.08) |
| $25,000 - $49,999 | 1.73***  (1.38-2.17) | 1.60***  (1.39-1.85) | 0.83  (0.50-1.40) | 1.44**  (1.15-1.79) | 1.79  (0.92-3.48) | 1.45*  (1.01-2.08) | 1.30  (0.34-4.93) | 0.90  (0.59-1.38) | 1.89***  (1.31-2.73) | 1.50**  (1.18-1.91) | 1.18  (0.85-1.63) | 1.12  (0.93-1.33) |
| $50,000 - $99,999 | 1.0 | 1.0 | 1.0 | 1.0 | 1.0 | 1.0 | 1.0 | 1.0 | 1.0 | 1.0 | 1.0 | 1.0 |
| $100,000 - $199,999 | 0.59***  (0.47-0.74) | 0.63***  (0.55-0.73) | 0.67*  (0.45-1.00) | 0.90  (0.74-1.10) | 0.54  (0.25-1.19) | 0.57**  (0.37-0.87) | 1.02  (0.30-3.53) | 0.67*  (0.44-1.03) | 0.76  (0.50-1.18) | 0.63**  (0.46-0.86) | 0.80  (0.60-1.08) | 0.96  (0.81-1.13) |
| $200,000 and above | 0.35***  (0.26-0.48) | 0.35***  (0.28-0.44) | 0.66  (0.41-1.06) | 0.72*  (0.54-0.95) | - | 0.44*  (0.24-0.83) | 0.75  (0.13-4.38) | 1.08  (0.65-1.81) | 0.50*  (0.26-0.97) | 0.72  (0.47-1.11) | 0.73  (0.49-1.07) | 0.80  (0.64-1.01) |
| **Employment status** |  |  |  |  |  |  |  |  |  |  |  |  |
| Employed | 1.0 | 1.0 | 1.0 | 1.0 | 1.0 | 1.0 | 1.0 | 1.0 | 1.0 | 1.0 | 1.0 | 1.0 |
| Not working | 0.66***  (0.56-0.79) | 0.80***  (0.72-0.89) | 0.17***  (0.11-0.25) | 0.16***  (0.13-0.20) | 1.11  (0.64-1.94) | 1.19  (0.90-1.56) | 1.43  (0.53-3.86) | 1.54**  (1.14-2.08) | 1.56**  (1.18-2.07) | 2.00***  (1.67-2.38) | 0.99  (0.79-1.25) | 1.02  (0.90-1.16) |
| **Health insurance**  **(ref: Private)** |  |  |  |  |  |  |  |  |  |  |  |  |
| Private | 1.0 | 1.0 | 1.0 | 1.0 | 1.0 | 1.0 | 1.0 | 1.0 | 1.0 | 1.0 | 1.0 | 1.0 |
| Medicare/Dual eligibility | **0.83^§^**  **(0.69-1.01)** | **1.10^§^**  **(0.95-1.27)** | 0.67  (0.40-1.12) | 0.48***  (0.33-0.70) | 1.97*  (1.07-3.62) | 1.21  (0.86-1.69) | 1.10  (0.30-4.02) | 0.98  (0.55-1.74) | 1.55**  (1.16-2.09) | 1.59***  (1.30-1.96) | 0.99  (0.77-1.28) | 0.93  (0.79-1.11) |
| Medicaid | 0.63**  (0.44-0.89) | 0.52***  (0.43-0.63) | 0.52  (0.26-1.03) | 0.74*  (0.55-0.98) | 2.93*  (1.28-6.70) | 1.75**  (1.19-2.58) | 1.37  (0.33-5.64) | 2.58***  (1.70-3.90) | 1.55*  (1.06-2.27) | 1.47***  (1.18-1.84) | 1.18  (0.81-1.74) | 1.09  (0.89-1.33) |
| Uninsured (including IHS only, single service plans) | 1.55  (0.96-2.50) | 2.33***  (1.69-3.22) | 0.90  (0.33-2.43) | 0.77  (0.46-1.29) | 3.68*  (1.17-11.57) | 1.38  (0.64-2.96) | 2.39  (0.42-13.48) | 2.73**  (1.33-5.59) | 1.58  (0.80-3.10) | 1.48  (0.97-2.27) | 1.24  (0.64-2.42) | 1.17  (0.79-1.73) |
| **Marital status** |  |  |  |  |  |  |  |  |  |  |  |  |
| Married | 1.0 | 1.0 | 1.0 | 1.0 | 1.0 | 1.0 | 1.0 | 1.0 | 1.0 | 1.0 | 1.0 | 1.0 |
| Divorced/Separated/Widowed | 1.09  (0.88-1.34) | 0.93  (0.82-1.05) | 0.90  (0.58-1.41) | 1.19  (0.99-1.45) | 0.98  (0.54-1.77) | 0.87  (0.65-1.18) | 0.26*  (0.07-0.98) | 0.67*  (0.46-0.97) | 1.79**  (1.34-2.40) | 1.41***  (1.17-1.69) | **1.38*^§^**  **(1.05-1.83)** | **0.92^§^**  **(0.80-1.06)** |
| Non-married | 0.81  (0.62-1.05) | 0.83*  (0.71-0.97) | 0.70  (0.44-1.11) | 1.07  (0.86-1.33) | 0.50  (0.22-1.14) | 1.16  (0.81-1.65) | **0.05***^§^**  **(0.01-0.24)** | **0.35***^§^**  **(0.23-0.53)** | 1.35  (0.96-1.91) | 1.26*  (1.01-1.58) | 1.36  (0.99-1.87) | 1.00  (0.84-1.19) |
| Living with partner | 1.24  (0.88-1.75) | 0.91  (0.73-1.14) | 0.76  (0.40-1.42) | 1.26  (0.95-1.69) | 0.72  (0.21-2.44) | 1.04  (0.61-1.78) | - | 0.69  (0.42-1.14) | 1.24  (0.73-2.12) | 1.03  (0.74-1.45) | **1.90**^§^**  **(1.27-2.85)** | **0.92^§^**  **(0.72-1.19)** |
| **Homeownership** |  |  |  |  |  |  |  |  |  |  |  |  |
| Owner | 1.0 | 1.0 | 1.0 | 1.0 | 1.0 | 1.0 | 1.0 | 1.0 | 1.0 | 1.0 | 1.0 | 1.0 |
| Renter | **1.10^§^**  **(0.89-1.34)** | **1.42***^§^**  **(1.26-1.61)** | 1.73**  (1.21-2.47) | 1.41***  (1.18-1.69) | 1.14  (0.62-2.09) | 0.82  (0.59-1.12) | **6.02***^§^**  **(2.19-16.52)** | **1.45*^§^**  **(1.04-2.04)** | 1.93***  (1.46-2.55) | 1.61***  (1.35-1.92) | **0.95^§^**  **(0.73-1.25)** | **1.31***^§^**  **(1.14-1.52)** |

Abbreviations: Adj., adjusted; CI, confidence interval; IHS, Indian health service; NH, non-Hispanic; NHPI, Native Hawaiian, Pacific Islander; OR, odds ratio.

^1^Delayed healthcare due to affordability reasons includes need of out-of-pocket payment, too high deductibles and cannot afford co-pay.

*P<0.05, **P<0.01, ***P<0.001.

^§^Coefficients are significantly different at 5% significance level (male vs female).

(-) Coefficients cannot be estimated.

**eTable S4.** Factors associated with various reasons for delayed healthcare, stratified by racial/ethnic background

|  | **Affordability^1^** | | | **Work** | | | **Elderly care** | | | **Childcare** | | | **Transportation** | | | **Nervous** | | |
| --- | --- | --- | --- | --- | --- | --- | --- | --- | --- | --- | --- | --- | --- | --- | --- | --- | --- | --- |
|  | **Adj. OR (95% CI)** | | | **Adj. OR (95% CI)** | | | **Adj. OR (95% CI)** | | | **Adj. OR (95% CI)** | | | **Adj. OR (95% CI)** | | | **Adj. OR (95% CI)** | | |
|  | **NHW** | **NHB** | **Hisp** | **NHW** | **NHB** | **Hisp** | **NHW** | **NHB** | **Hisp** | **NHW** | **NHB** | **Hisp** | **NHW** | **NHB** | **Hisp** | **NHW** | **NHB** | **Hisp** |
| **Age at survey** |  |  |  |  |  |  |  |  |  |  |  |  |  |  |  |  |  |  |
| 18-39 | 3.62***  (2.99-4.38) | 2.98***  (1.60-5.54) | 4.86***  (2.53-9.34) | 8.27***  (6.08-11.23) | 18.66***  (6.40-54.38) | 4.43**  (1.69-11.62) | 1.08  (0.59-1.98) | 2.48  (0.59-10.36) | 1.62  (0.36-7.22) | 103.10***  (46.79-227.19) | 18.36***  (3.70-91.16) | 44.89***  (7.79-258.66) | 6.11***  (4.60-8.12) | 4.36***  (2.28-8.36) | 4.03**  (1.76-9.23) | 5.38***  (4.38-6.61) | 3.30**  (1.52-7.18) | 4.14**  (1.90-9.04) |
| 40-64 | 2.87***  (2.55-3.22) | 2.13***  (1.50-3.02) | 2.45***  (1.49-4.04) | 3.70***  (2.87-4.76) | 3.61**  (1.46-8.96) | 2.34*  (1.02-5.34) | 1.91***  (1.40-2.61) | 1.63  (0.67-3.97) | 1.83  (0.63-5.32) | **10.62***^§^**  **(4.98-22.64)** | **2.03^§^**  **(0.50-8.25)** | 4.56  (0.92-22.62) | **3.39***^§^**  **(2.83-4.06)** | **1.82**^§^**  **(1.22-2.71)** | 3.11***  (1.73-5.59) | 2.47***  (2.15-2.84) | 2.56***  (1.59-4.10) | 2.05*  (1.12-3.74) |
| 65+ | 1.0 | 1.0 | 1.0 | 1.0 | 1.0 | 1.0 | 1.0 | 1.0 | 1.0 | 1.0 | 1.0 | 1.0 | 1.0 | 1.0 | 1.0 | 1.0 | 1.0 | 1.0 |
| **Biological sex** |  |  |  |  |  |  |  |  |  |  |  |  |  |  |  |  |  |  |
| Female | 1.0 | 1.0 | 1.0 | 1.0 | 1.0 | 1.0 | 1.0 | 1.0 | 1.0 | 1.0 | 1.0 | 1.0 | 1.0 | 1.0 | 1.0 | 1.0 | 1.0 | 1.0 |
| Male | 0.78***  (0.70-0.86) | 0.78  (0.57-1.09) | 0.91  (0.63-1.32) | **0.57***^§^**  **(0.47-0.69)** | **1.18^§^**  **(0.65-2.12)** | 0.55*  (0.32-0.95) | 0.55***  (0.41-0.75) | 0.52  (0.21-1.30) | 0.39  (0.14-1.07) | **0.13***^§†^**  **(0.06-0.26)** | **0.71^§^**  **(0.23-2.27)** | **0.48^†^**  **(0.18-1.29)** | 0.95  (0.81-1.11) | 0.97  (0.68-1.38) | 0.69  (0.42-1.15) | **0.56***^§^**  **(0.49-0.63)** | **0.91^§^**  **(0.60-1.39)** | 0.75  (0.47-1.19) |
| **Education attainment** |  |  |  |  |  |  |  |  |  |  |  |  |  |  |  |  |  |  |
| High school diploma or less | 1.00  (0.84-1.19) | 0.89  (0.56-1.41) | 0.84 (0.46-1.54) | 1.18  (0.87-1.59) | 1.41  (0.57-3.48) | 0.82  (0.38-1.76) | 1.05  (0.68-1.60) | 0.46  (0.14-1.54) | 1.06  (0.26-4.29) | 0.65  (0.35-1.18) | 0.54  (0.10-2.96) | 0.34  (0.09-1.22) | 1.37*  (1.06-1.76) | 1.40  (0.76-2.56) | 0.94  (0.41-2.11) | 1.10  (0.90-1.34) | 0.88  (0.48-1.63) | 1.66  (0.76-3.61) |
| Some college | 1.24**  (1.10-1.40) | 0.88  (0.58-1.34) | 0.97  (0.55-1.74) | 1.35**  (1.10-1.65) | 1.90  (0.86-4.23) | 0.88  (0.43-1.79) | 0.98  (0.69-1.37) | 0.84  (0.29-2.43) | 0.53  (0.12-2.39) | 0.77  (0.48-1.22) | 1.12  (0.25-5.06) | 0.37  (0.11-1.31) | 1.34**  (1.08-1.65) | 1.21  (0.67-2.19) | 0.78  (0.34-1.80) | 1.05  (0.91-1.22) | 0.86  (0.49-1.52) | 1.22  (0.56-2.67) |
| Bachelor | 1.09  (0.97-1.23) | 1.00  (0.64-1.56) | 1.31  (0.74-2.33) | **1.04^§^**  **(0.87-1.26)** | **2.34*^§^**  **(1.07-5.11)** | 1.11  (0.55-2.24) | 0.90  (0.64-1.25) | 0.83  (0.26-2.60) | 0.68  (0.15-3.10) | **1.06^†^**  **(0.71-1.58)** | 0.91  (0.18-4.48) | **0.14*^†^**  **(0.03-0.63)** | 1.16  (0.94-1.44) | 1.34  (0.71-2.53) | 0.63  (0.26-1.56) | 1.03  (0.90-1.18) | 1.45  (0.82-2.55) | 1.75  (0.81-3.77) |
| Master or more | 1.0 | 1.0 | 1.0 | 1.0 | 1.0 | 1.0 | 1.0 | 1.0 | 1.0 | 1.0 | 1.0 | 1.0 | 1.0 | 1.0 | 1.0 | 1.0 | 1.0 | 1.0 |
| **Annual household income** |  |  |  |  |  |  |  |  |  |  |  |  |  |  |  |  |  |  |
| Less than $25,000 | 1.76***  (1.48-2.10) | 1.45  (0.90-2.34) | 1.21  (0.68-2.15) | 1.23  (0.88-1.73) | 1.74  (0.72-4.16) | 1.25  (0.56-2.76) | 1.78**  (1.16-2.74) | 1.40  (0.39-4.98) | 2.65  (0.48-14.47) | 0.66  (0.37-1.17) | 1.94  (0.37-10.28) | 2.09  (0.48-9.15) | 3.05***  (2.40-3.88) | 2.46**  (1.29-4.67) | 1.50  (0.64-3.49) | 1.81***  (1.48-2.22) | 1.11  (0.58-2.13) | 1.43  (0.69-2.94) |
| $25,000 - $49,999 | 1.63***  (1.42-1.86) | 1.68*  (1.08-2.60) | 1.57  (0.94-2.63) | 1.21  (0.95-1.54) | 1.48  (0.68-3.12) | 1.94*  (1.01-3.74) | 1.49*  (1.04-2.13) | 1.18  (0.34-4.12) | 2.70  (0.50-14.64) | 0.67  (0.40-1.13) | 2.15  (0.49-9.40) | 2.00  (0.48-8.37) | 1.60***  (1.27-2.01) | 1.66  (0.87-3.18) | 1.07  (0.44-2.58) | 1.11  (0.93-1.32) | 1.13  (0.61-2.10) | 1.16  (0.58-2.32) |
| $50,000 - $99,999 | 1.0 | 1.0 | 1.0 | 1.0 | 1.0 | 1.0 | 1.0 | 1.0 | 1.0 | 1.0 | 1.0 | 1.0 | 1.0 | 1.0 | 1.0 | 1.0 | 1.0 | 1.0 |
| $100,000 - $199,999 | **0.63***^†^**  **(0.55-0.72)** | 0.42**  (0.23-0.77) | **0.27***^†^**  **(0.13-0.58)** | 0.87  (0.71-1.06) | 1.48  (0.61-3.59) | 0.54  (0.23-1.26) | 0.54**  (0.36-0.81) | 0.75  (0.14-4.06) | 0.73  (0.06-8.46) | 0.64  (0.42-1.03) | 0.76  (0.07-7.85) | 0.49  (0.05-4.95) | 0.68**  (0.52-0.90) | 0.72  (0.25-2.03) | - | 0.89  (0.76-1.03) | 1.47  (0.72-3.01) | 0.74  (0.31-1.75) |
| $200,000 and above | 0.36***  (0.30-0.44) | 0.07*  (0.01-0.54) | 0.21*  (0.05-0.96) | 0.69**  (0.53-0.91) | 1.53  (0.31-7.68) | 0.48  (0.10-2.24) | 0.29***  (0.14-0.58) | 1.19  (0.13-11.32) | - | 0.87  (0.50-1.52) | - | - | 0.64*  (0.43-0.95) | 0.54  (0.07-4.23) | 1.13  (0.14-9.68) | 0.75**  (0.61-0.92) | 0.64  (0.14-2.91) | - |
| **Employment status** |  |  |  |  |  |  |  |  |  |  |  |  |  |  |  |  |  |  |
| Employed | 1.0 | 1.0 | 1.0 | 1.0 | 1.0 | 1.0 | 1.0 | 1.0 | 1.0 | 1.0 | 1.0 | 1.0 | 1.0 | 1.0 | 1.0 | 1.0 | 1.0 | 1.0 |
| Not working | 0.76***  (0.68-0.84) | 0.73  (0.52-1.03) | 1.10  (0.75-1.62) | **0.13***^§†^**  **(0.10-0.16)** | **0.28***^§^**  **(0.15-0.53)** | **0.41**^†^**  **(0.24-0.71)** | 1.12  (0.84-1.49) | 1.23  (0.54-2.80) | 1.23  (0.51-2.99) | 1.58*  (1.11-2.24) | 1.17  (0.39-3.52) | 2.65*  (1.06-6.61) | **2.01***^§^**  **(1.68-2.41)** | **1.27^§‡^**  **(0.85-1.90)** | **2.60***^‡^**  **(1.53-4.41)** | 1.01  (0.89-1.14) | 0.92  (0.60-1.43) | 1.07  (0.66-1.73) |
| **Health insurance** |  |  |  |  |  |  |  |  |  |  |  |  |  |  |  |  |  |  |
| Private | 1.0 | 1.0 | 1.0 | 1.0 | 1.0 | 1.0 | 1.0 | 1.0 | 1.0 | 1.0 | 1.0 | 1.0 | 1.0 | 1.0 | 1.0 | 1.0 | 1.0 | 1.0 |
| Medicare/Dual eligibility | 1.00  (0.88-1.14) | 1.19  (0.80-1.77) | 0.62  (0.37-1.15) | 0.56**  (0.39-0.79) | 0.27*  (0.09-0.79) | 0.67  (0.27-1.67) | 1.30  (0.93-1.80) | 1.17  (0.38-3.60) | 1.20  (0.30-4.85) | 1.39  (0.73-2.66) | 0.37  (0.08-1.77) | 0.84  (0.18-3.88) | 1.60***  (1.32-1.94) | 1.20  (0.73-1.97) | 2.00  (0.93-4.32) | 0.91  (0.78-1.06) | 1.59  (0.92-2.76) | 1.16  (0.59-2.29) |
| Medicaid | 0.54***  (0.44-0.67) | 0.68  (0.43-1.08) | 0.47**  (0.29-0.78) | 0.72  (0.51-1.01) | 0.55  (0.26-1.18) | 0.74  (0.39-1.41) | 1.71*  (1.10-2.65) | 2.90  (0.98-8.58) | 1.48  (0.45-4.83) | **4.01***^§^**  **(2.40-6.70)** | **0.56^§^**  **(0.15-2.05)** | 1.55  (0.55-4.43) | 1.33*  (1.04-1.69) | 1.61  (0.97-2.66) | 2.46*  (1.23-4.91) | 1.16  (0.94-1.44) | 1.42  (0.80-2.54) | 0.78  (0.43-1.42) |
| Uninsured (including IHS only, single service plans) | **2.56***^§^**  **(1.84-3.58)** | **1.00^§^**  **(0.49-2.06)** | 2.51**  (1.28-4.93) | 0.79  (0.43-1.45) | 0.77  (0.26-2.26) | 0.60  (0.19-1.88) | 1.26  (0.49-3.21) | 1.52  (0.28-8.34) | 2.08  (0.45-9.54) | 3.82**  (1.55-9.40) | 1.27  (0.26-6.11) | 1.20  (0.20-7.08) | 1.52  (0.95-2.42) | 1.39  (0.70-2.78) | 2.20  (0.79-6.07) | 1.15  (0.76-1.76) | 1.39  (0.56-3.45) | 1.05  (0.42-2.65) |
| **Marital status** |  |  |  |  |  |  |  |  |  |  |  |  |  |  |  |  |  |  |
| Married | 1.0 | 1.0 | 1.0 | 1.0 | 1.0 | 1.0 | 1.0 | 1.0 | 1.0 | 1.0 | 1.0 | 1.0 | 1.0 | 1.0 | 1.0 | 1.0 | 1.0 | 1.0 |
| Divorced/Separated/Widowed | 0.97  (0.86-1.09) | 0.73  (0.52-1.04) | 0.90 (0.60-1.36) | 1.16  (0.95-1.43) | 1.19  (0.61-2.35) | 1.05  (0.61-1.81) | 1.01  (0.74-1.39) | 0.94  (0.40-2.25) | 0.55  (0.22-1.40) | 0.43***  (0.27-0.71) | 1.51  (0.42-5.40) | 0.92  (0.37-2.29) | 1.51***  (1.25-1.82) | 1.26  (0.81-1.95) | 1.62  (0.98-2.68) | **0.91^§^**  **(0.79-1.06)** | **1.57^§^**  **(0.96-2.58)** | 0.92  (0.55-1.55) |
| Non-married | 0.87  (0.75-1.02) | 0.60*  (0.40-0.89) | 0.76  (0.46-1.24) | 1.04  (0.82-1.31) | 0.95  (0.48-1.90) | 0.82  (0.43-1.57) | 1.22  (0.83-1.81) | 0.75  (0.29-1.93) | 0.76  (0.25-2.33) | 0.27***  (0.16-0.46) | 0.73  (0.19-2.77) | 0.20**  (0.06-0.67) | 1.41**  (1.12-1.76) | 0.98  (0.61-1.59) | 1.70  (0.93-3.13) | 1.02  (0.86-1.22) | 1.58  (0.93-2.69) | 1.58  (0.91-2.74) |
| Living with partner | **1.05^§^**  **(0.85-1.30)** | **0.45*^§^**  **(0.20-1.00)** | 0.58  (0.27-1.25) | 1.07  (0.78-1.46) | 1.73  (0.60-4.99) | 1.01  (0.41-2.49) | 1.33  (0.78-2.28) | - | 0.47  (0.06-3.77) | 0.52*  (0.29-0.94) | 1.47  (0.22-9.89) | 0.72  (0.17-3.03) | 1.01  (0.71-1.44) | 1.49  (0.71-3.13) | 1.62  (0.65-4.06) | 1.17  (0.93-1.48) | 1.41  (0.55-3.59) | 1.28  (0.56-2.93) |
| **Homeownership** |  |  |  |  |  |  |  |  |  |  |  |  |  |  |  |  |  |  |
| Owner | 1.0 | 1.0 | 1.0 | 1.0 | 1.0 | 1.0 | 1.0 | 1.0 | 1.0 | 1.0 | 1.0 | 1.0 | 1.0 | 1.0 | 1.0 | 1.0 | 1.0 | 1.0 |
| Renter | 1.36***  (1.20-1.53) | 1.11  (0.81-1.54) | 0.94  (0.64-1.38) | **1.57***^†^**  **(1.30-1.90)** | 1.76  (0.97-3.22) | **0.85^†^**  **(0.51-1.41)** | 0.85  (0.61-1.20) | 1.20  (0.51-2.83) | 0.91  (0.37-2.25) | 1.50*  (1.00-2.22) | 2.46  (0.72-8.40) | 4.08*  (1.11-14.99) | 1.67***  (1.40-2.00) | 1.63*  (1.09-2.44) | 1.39  (0.81-2.39) | 1.28***  (1.11-1.48) | 1.14  (0.74-1.76) | 0.96  (0.60-1.55) |

Abbreviations: Adj., adjusted; CI, confidence interval; IHS, Indian health service; Hisp, Hispanic; NHB, non-Hispanic Black; NHW, non-Hispanic White; OR, odds ratio.

^1^Delayed healthcare due to affordability reasons includes need of out-of-pocket payment, too high deductibles and cannot afford co-pay.

*P<0.05, **P<0.01, ***P<0.001.

^§^Coefficients are significantly different at 5% significance level (NHW vs NHB).

^†^Coefficients are significantly different at 5% significance level (NHW vs Hisp).

^‡^Coefficients are significantly different at 5% significance level (NHB vs Hisp).

(-) Coefficients cannot be estimated.

**eTable S5.** Factors associated with various reasons for delayed healthcare (affordability, work and elderly care), stratified by cancer type

|  | **Affordability^1^** | | | | **Work** | | | | **Elderly care** | | | |
| --- | --- | --- | --- | --- | --- | --- | --- | --- | --- | --- | --- | --- |
|  | **Adj. OR (95% CI)** | | | | **Adj. OR (95% CI)** | | | | **Adj. OR (95% CI)** | | | |
|  | **Breast** | **Prostate** | **Lung** | **Colo-rectal** | **Breast** | **Prostate** | **Lung** | **Colo-rectal** | **Breast** | **Prostate** | **Lung** | **Colo-rectal** |
| **Age at survey** |  |  |  |  |  |  |  |  |  |  |  |  |
| 18-39 | 5.05***  (3.25- 7.87) | - | 2.63  (0.33-21.06) | 5.35***  (2.01-14.26) | 5.54***  (2.80-10.96) | - | 232.70**  (4.09-13231.60) | 2.81  (0.46-17.13) | 1.32  (0.41-4.27) | - | - | 5.89  (0.40-87.57) |
| 40-64 | 2.83***  (2.23-3.59) | 2.04***  (1.38-3.03) | 2.20  (1.00-4.89) | 2.43**  (1.34-4.40) | 3.26***  (1.99-5.35) | 1.78  (0.71-4.48) | 4.48  (0.62-32.20) | 2.00  (0.55-7.33) | 1.19  (0.68-2.09) | 0.58  (0.09-3.59) | - | 2.24  (0.31-16.18) |
| 65+ | 1.0 | 1.0 | 1.0 | 1.0 | 1.0 | 1.0 | 1.0 | 1.0 | 1.0 | 1.0 | 1.0 | 1.0 |
| **Biological sex** |  |  |  |  |  |  |  |  |  |  |  |  |
| Female | 1.0 | 1.0 | 1.0 | 1.0 | 1.0 | 1.0 | 1.0 | 1.0 | 1.0 | 1.0 | 1.0 | 1.0 |
| Male | - | - | **2.29*^§§^**  **(1.11-4.72)** | **0.61*^§§^**  **(0.38-0.96)** | - | - | 0.44  (0.07-2.74) | 0.71  (0.32-1.58) | - | - | - | 0.62  (0.14-2.69) |
| **Race/ethnicity** |  |  |  |  |  |  |  |  |  |  |  |  |
| NH-White | 1.0 | 1.0 | 1.0 | 1.0 | 1.0 | 1.0 | 1.0 | 1.0 | 1.0 | 1.0 | 1.0 | 1.0 |
| NH-Black | 1.01  (0.74-1.39) | 0.86  (0.50-1.47) | 1.17  (0.34-4.04) | 1.46  (0.73-2.92) | 0.69  (0.39-1.22) | 1.82  (0.56-5.92) | 2.12  (0.09-50.42) | 1.11  (0.32-3.92) | 1.27  (0.62-2.60) | 0.38  (0.03-4.28) | - | 0.57  (0.05-7.02) |
| Hispanic | 0.83  (0.56-1.23) | 0.24*  (0.07-0.80) | 1.74  (0.36-8.50) | 0.78  (0.28-2.14) | 1.74*  (1.04-2.91) | 2.42  (0.60-9.81) | 0.15  (0.00-14.08) | 0.78  (0.14-4.23) | 0.94  (0.34-2.56) | - | - | - |
| NH-Asian/NHPI | 0.67  (0.37-1.21) | 0.43  (0.06-3.32) | 0.41  (0.05-3.64) | 1.62  (0.38-6.90) | 0.92  (0.43-1.97) | - | - | - | 3.29**  (1.36-8.01) | - | - | - |
| **Education attainment** |  |  |  |  |  |  |  |  |  |  |  |  |
| High school diploma or less | 0.90  (0.64-1.27) | 0.78  (0.41-1.47) | 0.34  (0.10-1.16) | 0.94  (0.44-2.02) | 1.73*  (1.02-2.92) | **0.32^¶^**  **(0.03-3.20)** | **30.01*^¶^**  **(1.16-779.78)** | 0.94  (0.19-4.59) | 0.95  (0.42-2.17) | 0.72  (0.07-7.82) | - | 0.92  (0.12-6.91) |
| Some college | 1.20  (0.94-1.54) | 1.13  (0.75-1.71) | 0.65  (0.22-1.88) | 0.91  (0.50-1.66) | 1.71  (0.79-1.74) | 1.64  (0.59-4.58) | 14.69  (0.70-309.64) | 1.28  (0.43-3.84) | 1.14  (0.62-2.12) | 1.75  (0.39-7.83) | - | 0.21  (0.02-2.53) |
| Bachelor | 1.16  (0.93-1.46) | 0.84  (0.57-1.24) | 1.44  (0.54-3.82) | 0.93  (0.52-1.66) | 1.14  (0.81-1.60) | 0.63  (0.20-1.94) | 2.13  (0.12-38.28) | 1.70  (0.61-4.75) | 0.87  (0.48-1.59) | 1.08  (0.25-4.61) | - | 1.41  (0.24-8.33) |
| Master or more | 1.0 | 1.0 | 1.0 | 1.0 | 1.0 | 1.0 | 1.0 | 1.0 | 1.0 | 1.0 | 1.0 | 1.0 |
| **Annual household income** |  |  |  |  |  |  |  |  |  |  |  |  |
| Less than $25,000 | 1.93***  (1.36-2.74) | 1.52  (0.82-2.83) | 1.83  (0.60-5.57) | 1.99  (0.84-4.74) | 1.74  (0.87-3.47) | 1.15  (0.16-8.35) | 5.11  (0.09-307.00) | 1.35  (0.26-6.98) | 2.19  (0.98-4.87) | 3.24  (0.29-35.85) | - | 2.92  (0.21-40.19) |
| $25,000 - $49,999 | 1.74***  (1.33-2.27) | 1.24  (0.77-1.98) | 1.19  (0.39-3.63) | 2.28*  (1.11-4.67) | 1.72*  (1.09-2.72) | 0.93  (0.21-4.17) | 3.38  (0.12-96.52) | 2.03  (0.66-6.25) | 1.41  (0.70-2.84) | 5.70  (0.99-32.88) | - | 3.05  (0.47-19.84) |
| $50,000 - $99,999 | 1.0 | 1.0 | 1.0 | 1.0 | 1.0 | 1.0 | 1.0 | 1.0 | 1.0 | 1.0 | 1.0 | 1.0 |
| $100,000 - $199,999 | **0.62***^‡^**  **(0.48-0.80)** | **0.45***^#^**  **(0.29-0.70)** | **0.29^§§^**  **(0.08-1.01)** | **1.51^‡#§§^**  **(0.78-2.93)** | 1.16  (0.79-1.71) | 0.32  (0.09-1.17) | 1.04  (0.09-12.46) | 1.23  (0.39-3.80) | 0.94  (0.46-1.89) | 2.53  (0.43-14.97) | - | - |
| $200,000 and above | 0.34***  (0.23-0.50) | 0.33***  (0.18-0.61) | 0.47  (0.10-2.12) | 0.48  (0.17-1.40) | 0.98  (0.60-1.59) | 0.85  (0.25-2.94) | 6.59  (0.23-188.48) | 0.19  (0.02-1.72) | 0.71  (0.25-1.98) | - | - | - |
| **Employment status** |  |  |  |  |  |  |  |  |  |  |  |  |
| Employed | 1.0 | 1.0 | 1.0 | 1.0 | 1.0 | 1.0 | 1.0 | 1.0 | 1.0 | 1.0 | 1.0 | 1.0 |
| Not working | 0.80*  (0.65-0.99) | 0.73  (0.51-1.05) | 0.50  (0.23-1.12) | 0.70  (0.42-1.19) | 0.16***  (0.11-0.26) | 0.13***  (0.04-0.41) | 0.06**  (0.01-0.49) | 0.07***  (0.02-0.29) | 1.48  (0.87-2.51) | 1.11  (0.26-4.75) | - | 0.74  (0.16-3.36) |
| **Health insurance**  **(ref: Private)** |  |  |  |  |  |  |  |  |  |  |  |  |
| Private | 1.0 | 1.0 | 1.0 | 1.0 | 1.0 | 1.0 | 1.0 | 1.0 | 1.0 | 1.0 | 1.0 | 1.0 |
| Medicare/Dual eligibility | 1.10  (0.84-1.45) | 0.99  (0.70-1.42) | 2.10  (0.86-5.13) | 0.73  (0.37-1.45) | 0.40*  (0.19-0.87) | 0.50  (0.15-1.66) | 0.24  (0.01-4.98) | 0.64  (0.11-3.77) | 1.17  (0.63-2.16) | 0.91  (0.25-3.35) | - | 6.01  (0.62-57.87) |
| Medicaid | 0.42***  (0.27-0.64) | 0.98  (0.38-2.58) | 0.95  (0.22-4.15) | 0.32*  (0.13-0.79) | 0.41*  (0.20-0.84) | - | 0.30  (0.00-24.58) | 1.37  (0.35-5.35) | 1.20  (0.50-2.88) | 9.38  (0.56-157.00) | - | 5.11  (0.56-46.93) |
| Uninsured (including IHS only, single service plans) | 1.44  (0.70-3.00) | 0.70  (0.32-1.57) | 0.24  (0.03-2.21) | 0.40  (0.06-2.73) | 0.50  (0.14-1.84) | - | - | 3.16  (0.30-33.77) | **2.19^‡^**  **(0.46-10.36)** | **-** | **-** | **115.60**^‡^**  **(5.07-2640.51)** |
| **Marital status** |  |  |  |  |  |  |  |  |  |  |  |  |
| Married | 1.0 | 1.0 | 1.0 | 1.0 | 1.0 | 1.0 | 1.0 | 1.0 | 1.0 | 1.0 | 1.0 | 1.0 |
| Divorced/Separated/Widowed | **0.81^§^**  **(0.64-1.02)** | **1.33^§^**  **(0.88-2.02)** | 1.03  (0.44-2.41) | 0.74  (0.40-1.38) | 0.96  (0.65-1.41) | 0.44  (0.12-1.68) | 0.10  (0.01-1.55) | 1.26  (0.45-3.53) | 0.92  (0.51-1.66) | 0.66  (0.14-3.10) | - | 0.91  (0.15-5.67) |
| Non-married | 0.66**  (0.48-0.89) | 1.00  (0.54-1.85) | 1.04  (0.32-3.38) | 1.18  (0.62-2.25) | **1.33^†^**  **(0.87-2.05)** | 0.47  (0.09-2.55) | **0.02^†^**  **(0.00-1.12)** | 1.18  (0.41-3.42) | 1.66  (0.85-3.23) | - | - | 2.78  (0.47-16.53) |
| Living with partner | **1.00^§^**  **(0.65-1.54)** | **3.01**^§¶#^**  **(1.47-6.15)** | **0.28^¶^**  **(0.03-2.51)** | **0.15^#^**  **(0.02-1.18)** | 1.23  (0.67-2.26) | 2.31  (0.50-10.59) | 0.24  (0.01-6.18) | 1.29  (0.24-7.00) | 0.87  (0.25-2.99) | - | - | - |
| **Homeownership** |  |  |  |  |  |  |  |  |  |  |  |  |
| Owner | 1.0 | 1.0 | 1.0 | 1.0 | 1.0 | 1.0 | 1.0 | 1.0 | 1.0 | 1.0 | 1.0 | 1.0 |
| Renter | **1.80***^§†^**  **(1.43-2.27)** | **0.74^§^**  **(0.45-1.22)** | **0.72^†^**  **(0.30-1.74)** | 1.39  (0.78-2.47) | 1.53*  (1.06-2.20) | **5.27***^§^**  **(1.99-13.95)** | **2.89^§^**  **(0.17-48.24)** | 1.77  (0.71-4.41) | 1.08  (0.58-1.99) | 0.67  (0.07-6.37) | - | 0.17  (0.02-1.37) |

Abbreviations: Adj., adjusted; CI, confidence interval; IHS, Indian health service; NH, non-Hispanic; NHPI, Native Hawaiian, Pacific Islander; OR, odds ratio.

^1^Delayed healthcare due to affordability reasons includes need of out-of-pocket payment, too high deductibles and cannot afford co-pay.

*P<0.05, **P<0.01, ***P<0.001.

^§^Coefficients are significantly different at 5% significance level (Breast vs Prostate).

^†^Coefficients are significantly different at 5% significance level (Breast vs Lung).

^‡^Coefficients are significantly different at 5% significance level (Breast vs Colorectal).

^¶^Coefficients are significantly different at 5% significance level (Prostate vs Lung).

^#^Coefficients are significantly different at 5% significance level (Prostate vs Colorectal).

^§§^Coefficients are significantly different at 5% significance level (Lung vs Colorectal).

(-) Coefficients cannot be estimated.

**eTable S6.** Factors associated with various reasons for delayed healthcare (childcare, transportation and nervous), stratified by cancer type

|  | **Childcare** | | | | **Transportation** | | | | **Nervous** | | | |
| --- | --- | --- | --- | --- | --- | --- | --- | --- | --- | --- | --- | --- |
|  | **Adj. OR (95% CI)** | | | | **Adj. OR (95% CI)** | | | | **Adj. OR (95% CI)** | | | |
|  | **Breast** | **Prostate** | **Lung** | **Colo-rectal** | **Breast** | **Prostate** | **Lung** | **Colo-rectal** | **Breast** | **Prostate** | **Lung** | **Colo-rectal** |
| **Age at survey** |  |  |  |  |  |  |  |  |  |  |  |  |
| 18-39 | 51.75***  (16.33-164.00) | - | - | - | 8.14***  (4.14-16.02) | - | - | 29.74***  (7.97-110.90) | **4.09***^‡^**  **(2.52-6.64)** | - | 3.87  (0.29-52.48) | **36.64***^‡^**  **(11.92-112.57)** |
| 40-64 | 5.95***  (2.15-16.47) | 4.88  (0.15-158.80) | - | - | 2.65***  (1.80-3.90) | 2.03*  (1.05-3.90) | 1.54  (0.54-4.36) | 3.64**  (1.55-8.56) | **2.08***^‡^**  **(1.59-2.72)** | **1.48^#^**  **(0.85-2.57)** | **1.96^§§^**  **(0.80-4.79)** | **6.39***^‡#§§^**  **(2.97-13.75)** |
| 65+ | 1.0 | 1.0 | 1.0 | 1.0 | 1.0 | 1.0 | 1.0 | 1.0 | 1.0 | 1.0 | 1.0 | 1.0 |
| **Sex** |  |  |  |  |  |  |  |  |  |  |  |  |
| Female | 1.0 | 1.0 | 1.0 | 1.0 | 1.0 | 1.0 | 1.0 | 1.0 | 1.0 | 1.0 | 1.0 | 1.0 |
| Male | - | - | - | - | - | - | **0.53^§§^**  **(0.19-1.52)** | **2.21*^§§^**  **(1.16-4.20)** | - | - | 0.81  (0.33-1.97) | 1.20  (0.72-2.03) |
| **Race/ethnicity** |  |  |  |  |  |  |  |  |  |  |  |  |
| NH-White | 1.0 | 1.0 | 1.0 | 1.0 | 1.0 | 1.0 | 1.0 | 1.0 | 1.0 | 1.0 | 1.0 | 1.0 |
| NH-Black | 1.14  (0.41-3.14) | 5.82  (0.22-151.14) | - | - | 1.41  (0.91-2.17) | 1.15  (0.56-2.36) | 1.82  (0.61-5.42) | 0.65  (0.25-1.70) | 0.67  (0.44-1.02) | 0.97  (0.46-2.06) | 0.45  (0.08-2.44) | 0.32*  (0.11-0.88) |
| Hispanic | 1.51  (0.55-4.14) | 14.10  (0.54-366.92) | - | - | 1.04  (0.60-1.80) | 0.82  (0.26-2.58) | 0.63  (0.06-6.38) | 0.51  (0.14-1.79) | 0.66  (0.40-1.06) | 0.64  (0.19-2.18) | 2.73  (0.34-22.03) | 0.65  (0.22-1.90) |
| NH-Asian/NHPI | 0.96  (0.21-4.47) | - | - | - | 1.26  (0.49-3.24) | 2.05  (0.43-9.75) | - | - | 0.56  (0.28-1.13) | 0.86  (0.11-6.53) | 0.75  (0.07-7.72) | - |
| **Education attainment** |  |  |  |  |  |  |  |  |  |  |  |  |
| High school diploma or less | 0.54  (0.18-1.59) | - | - | - | 2.28**  (1.31-3.95) | 1.75  (0.68-4.48) | 0.69  (0.18-2.64) | 1.07  (0.40-2.90) | 0.89  (0.60-1.32) | 1.43  (0.62-3.28) | 1.15  (0.30-4.45) | 1.05  (0.42-2.64) |
| Some college | 0.79  (0.36-1.75) | - | - | - | **2.08**^†‡^**  **(1.29-3.35)** | 1.74  (0.77-3.93) | **0.39^†^**  **(0.09-1.66)** | **0.57^‡^**  **(0.23-1.44)** | 0.93  (0.70-1.24) | 1.23  (0.67-2.28) | 1.97  (0.60-6.45) | 1.22  (0.59-2.53) |
| Bachelor | 0.60  (0.29-1.26) | - | - | - | **1.76*^‡^**  **(1.10-2.82)** | 1.61  (0.74-3.53) | 0.62  (0.14-2.76) | **0.59^‡^**  **(0.22-1.55)** | 0.97  (0.75-1.25) | 1.26  (0.74-2.14) | 0.58  (0.15-2.22) | 0.92  (0.45-1.88) |
| Master or more | 1.0 | 1.0 | 1.0 | 1.0 | 1.0 | 1.0 | 1.0 | 1.0 | 1.0 | 1.0 | 1.0 | 1.0 |
| **Annual household income** |  |  |  |  |  |  |  |  |  |  |  |  |
| Less than $25,000 | 1.65  (0.52-5.26) | - | - | - | 3.58***  (2.11-6.07) | **1.38^#^**  **(0.55-3.42)** | 3.09  (0.66-14.34) | **7.08**^#^**  **(1.99-25.14)** | 1.41  (0.92-2.15) | 1.29  (0.52-3.19) | 1.94  (0.57-6.54) | 2.82*  (1.01-7.89) |
| $25,000 - $49,999 | 0.88  (0.32-2.47) | - | - | - | 1.49  (0.89-2.47) | 2.27*  (1.05-4.87) | 1.32  (0.27-6.54) | 2.61  (0.71-9.54) | 0.94  (0.66-1.34) | 0.82  (0.37-1.82) | 4.71  (0.12-1.85) | 1.69  (0.65-4.39) |
| $50,000 - $99,999 | 1.0 | 1.0 | 1.0 | 1.0 | 1.0 | 1.0 | 1.0 | 1.0 | 1.0 | 1.0 | 1.0 | 1.0 |
| $100,000 - $199,999 | 0.85  (0.37-1.97) | 1.52  (0.07-31.25) | - | - | 0.84  (0.47-1.50) | 0.33  (0.09-1.18) | - | 1.36  (0.33-5.58) | 1.00  (0.75-1.33) | 0.73  (0.39-1.37) | **0.40^§§^**  **(0.11-1.46)** | **2.05^§§^**  **(0.83-5.02)** |
| $200,000 and above | 1.19  (0.45-3.16) | - | - | - | 1.02  (0.48-2.14) | 0.46  (0.10-2.15) | - | 1.42  (0.24-8.30) | **0.82^‡^**  **(0.56-1.19)** | **0.72^#^**  **(0.33-1.57)** | **0.27^§§^**  **(0.03-2.57)** | **3.63^‡#§§^**  **(1.30-10.10)** |
| **Employment status** |  |  |  |  |  |  |  |  |  |  |  |  |
| Employed | 1.0 | 1.0 | 1.0 | 1.0 | 1.0 | 1.0 | 1.0 | 1.0 | 1.0 | 1.0 | 1.0 | 1.0 |
| Not working | 3.12***  (1.69-5.75) | 0.24  (0.01-8.27) | - | - | 1.83**  (1.27-2.63) | 1.76  (0.87-3.58) | 2.23  (0.57-8.65) | 1.90  (0.84-4.29) | 1.21  (0.96-1.54) | **0.70^#^**  **(0.43-1.15)** | 0.75  (0.29-1.95) | **1.68^#^**  **(0.89-3.17)** |
| **Health insurance**  **(ref: Private)** |  |  |  |  |  |  |  |  |  |  |  |  |
| Private | 1.0 | 1.0 | 1.0 | 1.0 | 1.0 | 1.0 | 1.0 | 1.0 | 1.0 | 1.0 | 1.0 | 1.0 |
| Medicare/Dual eligibility | 0.37  (0.10-1.41) | 2.34  (0.04-126.60) | - | - | 1.34  (0.87-2.06) | 1.19  (0.61-2.32) | 1.11  (0.34-3.61) | 1.62  (0.63-4.15) | 0.91  (0.66-1.24) | 1.00  (0.60-1.66) | 0.41  (0.14-1.23) | 0.92  (0.40-2.11) |
| Medicaid | 1.17  (0.41-3.35) | - | - | - | 1.12  (0.66-1.88) | 3.02*  (1.15-7.94) | 1.39  (0.29-6.64) | 1.07  (0.38-3.03) | 1.12  (0.72-1.76) | 1.55  (0.49-4.90) | 0.69  (0.15-3.07) | 0.58  (0.22-1.51) |
| Uninsured (including IHS only, single service plans) | 5.52*  (1.47-20.72) | - | - | - | 1.78  (0.68-4.66) | 1.47  (0.29-7.36) | - | 2.26  (0.27-18.55) | 1.50  (0.63-3.58) | **0.64^#^**  **(0.08-5.04)** | 6.15  (0.69-54.67) | **10.38*^#^**  **(1.69-63.76)** |
| **Marital status** |  |  |  |  |  |  |  |  |  |  |  |  |
| Married | 1.0 | 1.0 | 1.0 | 1.0 | 1.0 | 1.0 | 1.0 | 1.0 | 1.0 | 1.0 | 1.0 | 1.0 |
| Divorced/Separated/Widowed | 0.51  (0.22-1.18) | 2.98  (0.16-57.02) | - | - | 1.97***  (1.33-2.92) | 2.18*  (1.11-4.26) | 1.80  (0.55-5.88) | 2.17  (0.91-5.15) | **0.81^†‡^**  **(0.62-1.08)** | **1.23^#^**  **(0.68-2.23)** | **2.88*^†^**  **(1.03-8.06)** | **3.48***^#‡^**  **(1.69-7.19)** |
| Non-married | 0.44  (0.17-1.14) | - | - | - | 1.95**  (1.21-3.14) | 1.93  (0.81-4.59) | 0.77  (0.14-4.24) | 2.42  (0.97-6.06) | **0.92^‡^**  **(0.65-1.30)** | 1.01  (0.40-2.55) | 1.44  (0.35-5.99) | **2.44*^‡^**  **(1.10-5.43)** |
| Living with partner | 0.32  (0.07-1.56) | - | - | - | 1.00  (0.42-2.35) | 3.01  (0.89-10.21) | 1.71  (0.23-12.57) | 1.02  (0.14-7.50) | 0.91  (0.55-1.53) | 2.66*  (1.03-6.84) | 1.80  (0.31-10.52) | 2.34  (0.70-7.81) |
| **Homeownership** |  |  |  |  |  |  |  |  |  |  |  |  |
| Owner | 1.0 | 1.0 | 1.0 | 1.0 | 1.0 | 1.0 | 1.0 | 1.0 | 1.0 | 1.0 | 1.0 | 1.0 |
| Renter | 2.30*  (1.13-4.70) | 1.19  (0.05-30.47) | - | - | 1.28  (0.88-1.85) | 1.98*  (1.02-3.85) | 1.78  (0.64-4.99) | 1.31  (0.58-2.94) | **1.74***^§†^**  **(1.32-2.28)** | **0.81^§^**  **(0.41-1.62)** | **0.30*^†^**  **(0.10-0.93)** | 0.94  (0.47-1.88) |

Abbreviations: Adj., adjusted; CI, confidence interval; IHS, Indian health service; NH, non-Hispanic; NHPI, Native Hawaiian, Pacific Islander; OR, odds ratio.

*P<0.05, **P<0.01, ***P<0.001.

^§^Coefficients are significantly different at 5% significance level (Breast vs Prostate).

^†^Coefficients are significantly different at 5% significance level (Breast vs Lung).

^‡^Coefficients are significantly different at 5% significance level (Breast vs Colorectal).

^¶^Coefficients are significantly different at 5% significance level (Prostate vs Lung).

^#^Coefficients are significantly different at 5% significance level (Prostate vs Colorectal).

^§§^Coefficients are significantly different at 5% significance level (Lung vs Colorectal).

(-) Coefficients cannot be estimated.
